# Motivational and Contextual Factors Across Sport Entry, Current Participation, Maintenance, and Withdrawal Among People with Physical Disabilities: A Systematic Review

**DOI:** 10.3390/sports14100457

**Published:** 2026-10-14

**Authors:** Gül Eda Burmaoğlu, Uğur Abakay

**Affiliations:** 1 Department of Sport Management, Faculty of Sport Sciences, Atatürk University, 25240 Erzurum, Türkiye; 2 Department of Physical Education and Sports, Faculty of Sport Sciences, Gaziantep University, 27310 Gaziantep, Türkiye; uabakay@gantep.edu.tr

**Keywords:** sport participation, physical disability, Paralympic sport, motivation, self-determination theory, barriers and facilitators, dropout, participation trajectory, rehabilitation, systematic review

## Abstract

Background: Sport participation can benefit people with physical disabilities, but whether they start, continue, or withdraw depends on motivational and contextual conditions. This systematic review aimed to synthesize evidence on sport entry, current participation, maintenance-related processes, and withdrawal or nonparticipation. Methods: Following the Preferred Reporting Items for Systematic Reviews and Meta-Analyses (PRISMA) 2020 guidelines, we searched Scopus, Web of Science, and PubMed/MEDLINE up to 5 September 2026. Two reviewers independently screened, verified, extracted, and appraised studies using the Mixed Methods Appraisal Tool (MMAT); the review was not prospectively registered. Results: Across the 106 included studies, accessibility, social support, and cost were frequently examined, whereas motivational and contextual factors were rarely examined together. Sixty-one studies (57.5%) described currently active participants cross-sectionally. Thirteen (12.3%) addressed maintenance-related processes, but only five (4.7%) provided behavioral evidence of persistence. Entry was addressed in 29 studies (27.4%) and withdrawal or nonparticipation in 11 (10.4%); these categories overlapped. Sixty-one studies (57.5%) reported no explicit theoretical framework. Conclusions: The evidence describes current participants considerably better than it explains how participation is initiated, sustained, or discontinued. Practice should address motivation together with access, social support, cost, equipment, coaching, and organizational conditions.

## 1. Introduction

Participation in sport is widely recognized as an important means of promoting physical, psychological, and social well-being among people with physical disabilities [1]. Meta-analytic evidence shows that physical activity yields meaningful benefits for this population in cardiovascular fitness, musculoskeletal fitness, cardiometabolic risk factors, and brain and mental health outcomes [1]. Physical activity has also been consistently associated with higher self-esteem, greater social integration, and better quality of life [2]. Article 30(5) of the United Nations Convention on the Rights of Persons with Disabilities explicitly recognizes the right of people with disabilities to participate in recreation, leisure, and sport on an equal basis with others [3]. The Paralympic Movement has grown from 400 athletes representing 23 countries at the first Paralympic Games in Rome in 1960 to approximately 4400 athletes from 169 delegations at Paris 2024 [4,5], reflecting an expanding, although still uneven, global infrastructure for such participation.

Despite this recognition, global evidence indicates that people with disabilities are approximately 16–62% less likely than people without disabilities to meet physical activity guidelines, with the range reflecting variation across disability groups and study contexts [1]. This estimate concerns overall physical activity rather than organized sport and should not be interpreted as a measure of sport-participation prevalence. People with physical disabilities who do take part in sport face a distinctive set of challenges throughout their sporting careers [6]. Their engagement is shaped by interacting individual, social, and structural conditions, ranging from motivation and psychological need satisfaction to facility accessibility, adapted equipment, transportation, coaching expertise, and disability-specific policy [6]. Understanding participation is therefore not only a matter of individual motivation but also of the broader ecological context in which sport opportunities are created, sustained, or closed off. Accordingly, social-ecological models have been used to organize barriers and facilitators to physical activity for people with disabilities across intrapersonal, interpersonal, organizational or institutional, and environmental or policy levels [1,7]. In this review, sport is distinguished from the broader construct of physical activity as an organized, rule-governed form of movement. Sport is typically structured around a recognized activity or competition (e.g., wheelchair basketball, para-athletics, or adapted swimming) and is often embedded in a club, team, or program. General exercise or physical activity interventions without such an organized or adapted-sport context fall outside the eligibility criteria of this review (Section 2.2).

### 1.1. Sport Participation Processes and Evidence Categories

Drawing on the organized-sport dropout literature [8] and the Quality Parasport Participation Framework [9], this review initially conceptualized participation in terms of three temporal processes: entry, maintenance or continuation, and withdrawal or dropout. During data verification, however, it became clear that many studies sampled people who were already active at a single time point and provided no evidence that participation had recently begun, was being sustained, or was approaching discontinuation. Current or general participation was therefore retained as a cross-sectional evidence category rather than as a fourth sequential stage. Maintenance-related evidence was further divided into indirect or prospective indicators, such as intention or commitment, and observed behavioral persistence over time. This approach preserves a participation-process perspective without implying that the included studies tracked participation longitudinally. Disability-specific frameworks support this distinction: the Quality Parasport Participation Framework identifies conditions in the physical, activity, and social environments that shape the quality of parasport experiences and continued participation [9], suggesting that relevant factors may differ across entry, current participation, continuation, and withdrawal.

Self-Determination Theory, including its sub-theory of basic psychological needs (autonomy, competence, and relatedness) [10], and socio-ecological models of health behavior [7] have both been applied, to varying degrees, to explain sport participation among people with physical disabilities. For example, qualitative work with elite athletes with physical disabilities has shown that participants attribute their sport engagement to self-determination constructs as well as to the need to overcome specific barriers such as cost, time constraints, and lack of opportunity [11]. However, these frameworks have typically been applied in isolation and to a single part of the participation process, most commonly initial engagement or general participation frequency. They have rarely been used to build a cumulative picture of how motivational and contextual factors operate across entry, maintenance, and withdrawal.

### 1.2. Rationale for the Present Review

Several reviews have addressed physical activity or sport participation in specific subgroups of people with physical disabilities (e.g., spinal cord injury, cerebral palsy, or limb loss) or in specific settings (e.g., school-based or community-based programs) [6]. Nevertheless, the literature remains fragmented in three ways. First, motivational factors (e.g., intrinsic motivation, self-determination, and athletic identity) and contextual factors (e.g., social support, accessibility, and institutional policy) have generally been studied separately, despite theoretical and empirical evidence that they interact [6]. Second, most evidence addresses only one part of the participation process, which makes it difficult to determine which factors are specific to entry, current participation, continuation, or withdrawal and which operate across these processes. Recent reviews illustrate this fragmentation from different angles. One systematic literature review found that participation remains an ambiguous and inconsistently operationalized construct in organized-sport research on disability [12]. Another synthesized barriers to mainstream-sport participation without distinguishing which participation process those barriers affected [13]. A third examined the psychometric properties of participation-assessment instruments for children and youth with disabilities but did not address the motivational or contextual factors those instruments are used to study [14]. None of these reviews distinguished cross-sectional evidence on current participation from direct evidence of entry, persistence, or dropout. Third, to our knowledge, no previous systematic review has synthesized this evidence while transparently appraising the methodological quality of both quantitative and qualitative studies within a single design-inclusive appraisal framework.

Addressing this gap matters for both theory and practice. Theoretically, a synthesis organized around participation processes and clearly defined evidence categories can show whether existing motivational frameworks generalize across entry, current participation, maintenance-related processes, and withdrawal, or whether they require process-specific extension. Practically, coaches, adapted physical activity practitioners, rehabilitation professionals, and policymakers need an integrated understanding of which factors matter at different points in participation. Such an understanding allows interventions and structural supports to be targeted more precisely at sport entry, continued participation, and dropout prevention.

### 1.3. Research Questions

To address this gap, the review was guided by the following research questions:

RQ1. What motivational and contextual factors are associated with sport participation among people with physical disabilities?

RQ2. How are these factors represented across evidence relating to sport entry and initiation, current or general participation, maintenance-related processes, and withdrawal, dropout, or nonparticipation?

RQ3. To what extent have motivational and contextual factors been examined within the same studies?

RQ4. What methodological, theoretical, geographical, and participation-process gaps characterize the current evidence base?

By mapping motivational and contextual factors across participation processes and clearly defined evidence categories, this review aimed to provide a structured, theory-informed synthesis that supports future research and applied efforts to promote sustained sport participation among people with physical disabilities.

## 2. Materials and Methods

### 2.1. Study Design and Registration

This study was conducted as a systematic review and reported in accordance with the Preferred Reporting Items for Systematic Reviews and Meta-Analyses (PRISMA) 2020 statement [15]. The review addressed focused questions about motivational and contextual factors associated with sport participation in a predefined population. It used explicit eligibility criteria, reproducible database searches, independent dual screening and extraction, and formal design-specific methodological appraisal, and these features were the basis for retaining the systematic-review designation. We acknowledge, however, that the synthesis also maps the distribution of evidence across participation processes and that this descriptive mapping component overlaps with features commonly associated with scoping reviews. Before screening began, the authors specified the eligibility criteria (Table 1), the database search strategy (Table 2), the screening and exclusion codes (Table 3 and Table 4), the data-extraction fields, and the MMAT appraisal approach. The review was not prospectively registered in a review registry, and no independently timestamped standalone protocol was produced; post hoc analytical modifications are therefore identified explicitly throughout the manuscript.

### 2.2. Eligibility Criteria

Because this review addresses motivational and contextual correlates of sport participation rather than the effects of a defined intervention, the “Intervention” component of the PICOS framework was interpreted as the exposure or phenomenon of interest, consistent with reviews of etiological or associative (non-intervention) evidence. PICOS was nevertheless retained because the eligible evidence base included both observational studies and genuine intervention studies (e.g., randomized controlled trials of structured sport programs; Table 5). A comparator-inclusive, design-flexible framework was therefore considered more appropriate than an exposure-specific framework that assumes an exclusively non-interventional evidence base. The eligibility criteria are summarized in Table 1.

**Table 1:** PICOS eligibility criteria.

PICOS Element	Definition Applied in This Review
Population (P)	Individuals with physical, motor, or mobility disabilities, including but not limited to spinal cord injury, limb loss/amputation, limb deficiency, cerebral palsy, spina bifida, and related physical/motor/mobility impairments. Studies involving participants with exclusively visual, hearing, or intellectual disabilities were excluded. Mixed-disability samples were eligible only when findings for participants with physical disabilities could be separately extracted or clearly attributed.
Exposure/Phenomenon of interest (I)	Participation in organized, adaptive/adapted, disability-specific, recreational, competitive, or elite (Para)sport. The core phenomenon of interest comprised individual, psychological, social, and environmental factors associated with sport entry, current participation, maintenance, commitment/persistence, withdrawal/dropout, or nonparticipation.
Comparison (C)	No comparator was required for eligibility. Where present, comparisons could involve participants versus nonparticipants, continuing versus withdrawn athletes, different sport disciplines, disability groups, participation levels, or intervention/control groups. Qualitative studies were not required to include a comparator.
Outcome (O)	Motivation, motives/reasons, barriers, facilitators, determinants, social/peer/family support, accessibility, sport opportunities, inclusion, commitment, persistence, continuation, dropout, withdrawal, and nonparticipation related to sport participation. Sport-specific motivational constructs (e.g., self-determination, basic psychological needs) were also eligible.
Study design (S)	Primary empirical quantitative, qualitative, and mixed-methods studies. Intervention studies were eligible if they reported outcomes directly related to sport participation. Systematic/narrative reviews, meta-analyses, editorials, perspectives/commentaries, protocols, book chapters, conference abstracts or presentation-only records without a full peer-reviewed empirical article, and case reports/single-case studies were excluded.

### 2.3. Information Sources and Search Strategy

Three electronic databases were searched: Scopus, Web of Science Core Collection, and PubMed/MEDLINE. These databases were selected because together they provide multidisciplinary, citation-indexed, biomedical, rehabilitation, and sport-related coverage, and because they allow the search strategy to be reproduced across sources. No lower publication-date limit was applied (i.e., records were retrieved from database inception to the final search date), and no language restriction was used. The final searches in all three databases were completed on 5 September 2026. Table 2 reports the number of records retrieved from each database, and the full Boolean search strings are reproduced below. These strings were checked against the original saved search sessions and confirmed to match the searches as executed. SPORTDiscus, PsycINFO, CINAHL, and Embase were not searched, and no backward or forward citation chasing or related-article searching was undertaken during the original review process; these scope decisions are addressed in the Limitations section.

**Table 2:** Search results by database.

Database	Records
Scopus	829
Web of Science	784
PubMed/MEDLINE	936
**Total**	**2549**

The full Boolean search strings for each database are reproduced below.

Scopus:

TITLE-ABS-KEY((“physical disabilit*” OR “physical impairment*” OR “physically disabled” OR “motor disabilit*” OR “motor impairment*” OR “mobility impairment*” OR “spinal cord injur*” OR amput* OR “limb deficien*” OR “cerebral palsy” OR “spina bifida”) AND (athlete* OR sport* OR parasport* OR “para sport*” OR Paralympic* OR “adaptive sport*” OR “adapted sport*” OR “disability sport*” OR “wheelchair sport*”) AND (motivat* OR “participation motive*” OR “participation reason*” OR “participation factor*” OR “participation determinant*” OR “sport participation” OR “sports participation” OR barrier* OR facilitat* OR “social support” OR “peer support” OR “family support” OR “access to sport” OR “sport access*” OR “sport opportunit*” OR “participation opportunit*” OR “sport inclusion” OR “sport continuation” OR “sport persistence” OR “sport commitment” OR “sport dropout” OR “sport withdrawal” OR “sport discontinuation” OR “sport nonparticipation” OR “sport non-participation” OR “self-determination” OR “self determination” OR “basic psychological need*”))

Web of Science:

TS = ((“physical disabilit*” OR “physical impairment*” OR “physically disabled” OR “motor disabilit*” OR “motor impairment*” OR “mobility impairment*” OR “spinal cord injur*” OR amput* OR “limb deficien*” OR “cerebral palsy” OR “spina bifida”) AND (athlete* OR sport* OR parasport* OR “para sport*” OR Paralympic* OR “adaptive sport*” OR “adapted sport*” OR “disability sport*” OR “wheelchair sport*”) AND (motivat* OR “participation motive*” OR “participation reason*” OR “participation factor*” OR “participation determinant*” OR “sport participation” OR “sports participation” OR barrier* OR facilitat* OR “social support” OR “peer support” OR “family support” OR “access to sport” OR “sport access*” OR “sport opportunit*” OR “participation opportunit*” OR “sport inclusion” OR “sport continuation” OR “sport persistence” OR “sport commitment” OR “sport dropout” OR “sport withdrawal” OR “sport discontinuation” OR “sport nonparticipation” OR “sport non-participation” OR “self-determination” OR “self determination” OR “basic psychological need*”))

PubMed/MEDLINE:

(“Disabled Persons”[Mesh] OR “Amputees”[Mesh] OR “Spinal Cord Injuries”[Mesh] OR “Cerebral Palsy”[Mesh] OR “physical disability”[Title/Abstract] OR “physical disabilities”[Title/Abstract] OR “physical impairment”[Title/Abstract] OR “physical impairments”[Title/Abstract] OR “physically disabled”[Title/Abstract] OR “motor disability”[Title/Abstract] OR “motor disabilities”[Title/Abstract] OR “motor impairment”[Title/Abstract] OR “motor impairments”[Title/Abstract] OR “mobility disability”[Title/Abstract] OR “mobility disabilities”[Title/Abstract] OR “mobility impairment”[Title/Abstract] OR “mobility impairments”[Title/Abstract] OR “spinal cord injury”[Title/Abstract] OR “spinal cord injuries”[Title/Abstract] OR paraplegi*[Title/Abstract] OR tetraplegi*[Title/Abstract] OR quadriplegi*[Title/Abstract] OR amput*[Title/Abstract] OR “limb deficiency”[Title/Abstract] OR “limb deficiencies”[Title/Abstract] OR “limb loss”[Title/Abstract] OR “lower limb amputation”[Title/Abstract] OR “upper limb deficiency”[Title/Abstract] OR “cerebral palsy”[Title/Abstract] OR “spina bifida”[Title/Abstract] OR “musculoskeletal disability”[Title/Abstract] OR “musculoskeletal disabilities”[Title/Abstract] OR “wheelchair user”[Title/Abstract] OR “wheelchair users”[Title/Abstract] OR “wheelchair athlete”[Title/Abstract] OR “wheelchair athletes”[Title/Abstract] OR “Paralympic athlete”[Title/Abstract] OR “Paralympic athletes”[Title/Abstract] OR “para athlete”[Title/Abstract] OR “para athletes”[Title/Abstract]) AND (“Sports”[Mesh] OR athlete*[Title/Abstract] OR sport*[Title/Abstract] OR parasport*[Title/Abstract] OR “para sport”[Title/Abstract] OR “para sports”[Title/Abstract] OR Paralympic*[Title/Abstract] OR “adaptive sport”[Title/Abstract] OR “adaptive sports”[Title/Abstract] OR “adapted sport”[Title/Abstract] OR “adapted sports”[Title/Abstract] OR “disability sport”[Title/Abstract] OR “disability sports”[Title/Abstract] OR “wheelchair sport”[Title/Abstract] OR “wheelchair sports”[Title/Abstract]) AND (motivat*[Title/Abstract] OR “participation motive”[Title/Abstract] OR “participation motives”[Title/Abstract] OR “participation reason”[Title/Abstract] OR “participation reasons”[Title/Abstract] OR “participation factor”[Title/Abstract] OR “participation factors”[Title/Abstract] OR “participation determinant”[Title/Abstract] OR “participation determinants”[Title/Abstract] OR “sport participation”[Title/Abstract] OR “sports participation”[Title/Abstract] OR barrier*[Title/Abstract] OR facilitat*[Title/Abstract] OR “social support”[Title/Abstract] OR “peer support”[Title/Abstract] OR “family support”[Title/Abstract] OR “access to sport”[Title/Abstract] OR “sport access”[Title/Abstract] OR “sport accessibility”[Title/Abstract] OR “sport opportunity”[Title/Abstract] OR “sport opportunities”[Title/Abstract] OR “participation opportunity”[Title/Abstract] OR “participation opportunities”[Title/Abstract] OR “sport inclusion”[Title/Abstract] OR “sport continuation”[Title/Abstract] OR “sport persistence”[Title/Abstract] OR “sport commitment”[Title/Abstract] OR “sport dropout”[Title/Abstract] OR “sport withdrawal”[Title/Abstract] OR “sport discontinuation”[Title/Abstract] OR “sport nonparticipation”[Title/Abstract] OR “sport non-participation”[Title/Abstract] OR “self-determination”[Title/Abstract] OR “self determination”[Title/Abstract] OR “basic psychological need”[Title/Abstract] OR “basic psychological needs”[Title/Abstract])

As a post hoc search-sensitivity check, we examined six sentinel sport-specific primary studies cited in the earlier systematic review by Jaarsma et al. [6]. These studies clearly matched the core eligibility domains of the present review: [16,17,18,19,20,21]. All six had been retrieved by the executed searches and were retained in the final 106-study corpus. This benchmark check was intended to detect obvious search failure for established, highly relevant studies; it does not replace searching additional specialist databases or formal citation chasing. In addition, although the eligibility criteria permitted recreational sport, the executed search relied on broad sport* and adapted, adaptive, and parasport terminology rather than on explicit recreation or leisure terms. Similarly, explicit adherence, retention, engagement, uptake, initiation, and maintenance terms were not included. Some records using these concepts could still have been retrieved through the broader sport and participation terms, but this terminology mismatch may have reduced search sensitivity and is therefore treated as a limitation.

### 2.4. Study Selection Process

**Figure 1:**
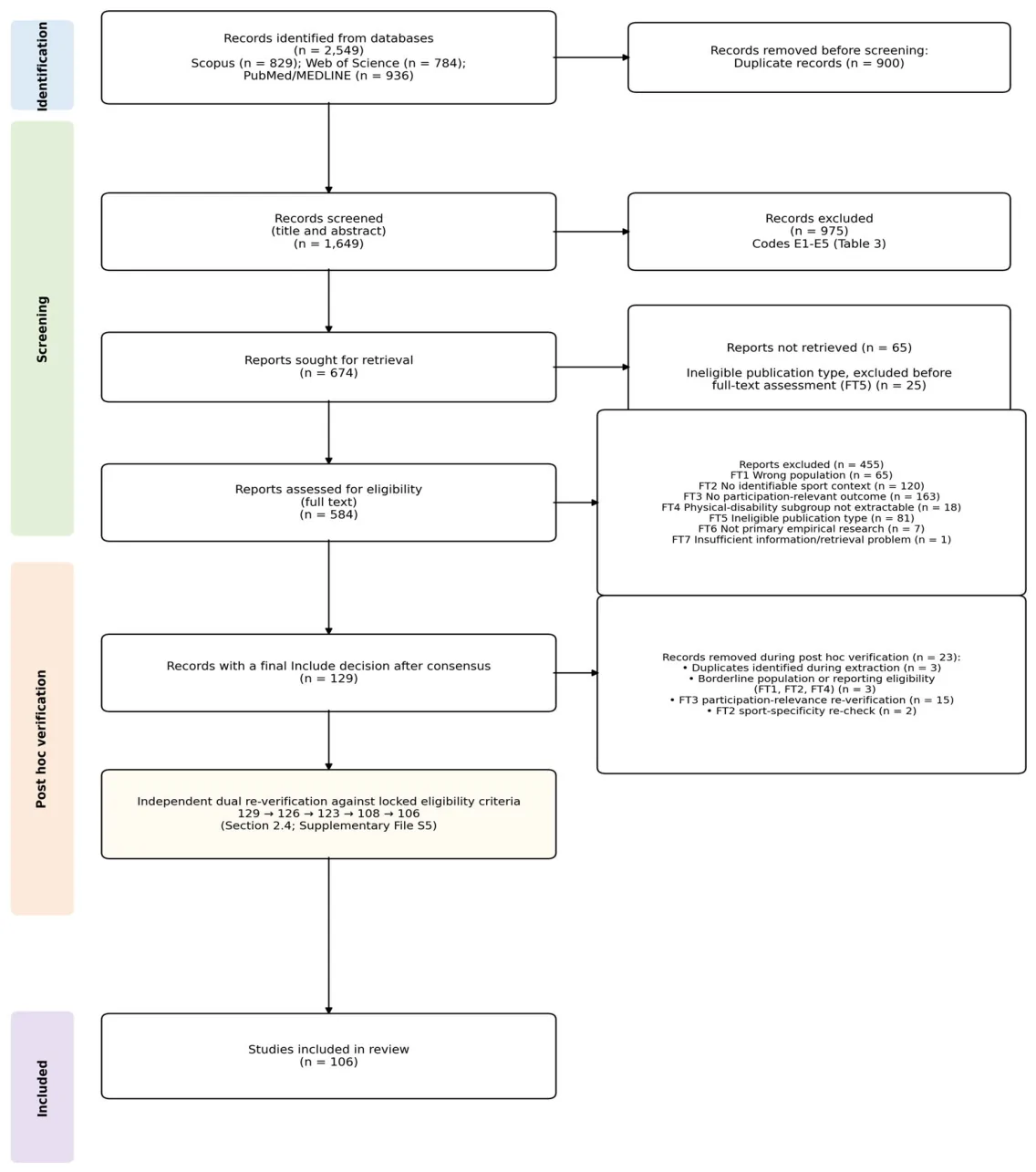
PRISMA 2020 flow diagram of the identification, screening, eligibility, post hoc verification, and inclusion process. Full-text exclusions are reported by FT1–FT7 code and count; code definitions are provided in Table 4.

Study selection followed the stages of the PRISMA 2020 flow diagram [15] (Figure 1). In the identification phase, 2549 records were retrieved (PubMed = 936, Scopus = 829, Web of Science = 784). After 900 duplicate records were removed, 1649 unique records remained for title and abstract screening.

#### 2.4.1. Title/Abstract Screening

Two reviewers independently screened titles and abstracts and classified each record as Include, Exclude, or Unclear according to five predefined exclusion codes (Table 3). Records were classified as Unclear when the title and abstract did not provide enough information for a confident decision; to minimize the risk of erroneous exclusion, these records were forwarded to full-text assessment rather than excluded.

**Table 3:** Title/abstract exclusion criteria (E1–E5).

Code	Criterion	Application
E1	Wrong population	No physical/motor/mobility disability population (e.g., non-disabled athletes, visual impairment, intellectual disability).
E2	No identifiable sport context	General physical activity, exercise, fitness, rehabilitation, physical education, or daily mobility, without an identifiable organized/adaptive sport context.
E3	No participation-relevant factor/outcome	Sport context present, but the study does not address motivation, entry, continuation, withdrawal, nonparticipation, or barriers/facilitators to participation (e.g., performance, injury, physiology, quality of life, or mental health as the primary outcome).
E4	Physical-disability subgroup not extractable	Mixed-disability sample in which the physical-disability subgroup cannot be separately evaluated.
E5	Ineligible publication type	Review, systematic review, editorial, commentary/perspective, protocol, book chapter, conference abstract or presentation-only record without a full peer-reviewed empirical article, case report, etc.

The reviewers agreed on 1332 of the 1649 screened records (raw agreement = 80.8%; Cohen’s κ = 0.62, indicating substantial agreement [22]). The 317 discordant records were initially resolved through a structured consensus procedure. During a subsequent data-verification pass, the two independent screening files were cross-checked against the original consensus workbook. This check showed that the workbook was incomplete as a documentation record: six entries did not preserve the reviewers’ original concordant decision, and 21 genuinely discordant records lacked a documented final consensus decision. Rather than overwriting the original workbook, both reviewers jointly re-examined these 27 records against the predefined title and abstract criteria and recorded a final decision for each in a separate verification record. After these corrections, 975 records were excluded and 674 were included for full-text retrieval. The original consensus workbook and the completed 27-record verification record have been retained in the internal audit archive.

#### 2.4.2. Full-Text Retrieval and Screening

Full-text retrieval was attempted for all 674 records included at the title and abstract stage. Supplementary retrieval efforts yielded full texts for 14 journal records that were initially unavailable, and these were added to both reviewers’ full-text assessment files. Despite e-mail requests to corresponding authors, to which no responses were received, 65 journal reports remained unavailable and were classified as not retrieved. A further 25 records were ineligible publication types that had not been fully removed by the original document-type limits (5 books, 10 book chapters, 8 conference papers, 1 web page, and 1 WHO report); these were excluded before formal dual eligibility assessment (reason: FT5).

The remaining 584 records were independently assessed in full text by the same two reviewers using seven predefined exclusion codes (Table 4).

**Table 4:** Full-text exclusion criteria (FT1–FT7).

Code	Criterion	Application
FT1	Wrong population	Original data source is not an eligible physical/motor/mobility disability population (e.g., coaches, healthcare professionals, organizations, non-disabled participants, animal studies).
FT2	No identifiable sport context	General physical activity, exercise, rehabilitation, fitness, mobility, or physical education, without a separate, identifiable organized/adaptive sport context.
FT3	No participation-relevant factor/outcome	Appropriate sport context and population present, but sport participation functions only as an exposure/background variable; the primary outcome is, e.g., quality of life, mental health, identity, injury, physiology, performance, fitness, or cognitive function.
FT4	Physical-disability subgroup not extractable	Physical-disability participants embedded in a mixed sample whose results cannot be separately extracted or reliably attributed.
FT5	Ineligible publication type	Review, systematic review, meta-analysis, perspective, editorial/commentary, protocol, book chapter, conference abstract or presentation-only record without a full peer-reviewed empirical article, case report/single-case study, etc.
FT6	Not primary empirical research	Publication type otherwise eligible, but the work does not present primary empirical participant-level evidence (conceptual, framework, technical/design, or similar).
FT7	Insufficient information/retrieval problem	Full text could not be obtained, or the retrieved material was insufficient to support a reliable eligibility decision.

The reviewers agreed on 578 of the 584 full-text records (124 included by both and 454 excluded by both; raw agreement = 98.97%; Cohen’s κ = 0.97, indicating almost perfect agreement [22]). In all six discordant cases, one reviewer had selected Include and the other Exclude; consensus discussion resulted in five additional inclusions and one confirmed exclusion (reason: FT3). In total, 129 records received a final Include decision. For 453 of the 454 records excluded by both reviewers, the same primary exclusion code was assigned. The single discrepancy (FT2 vs. FT3) was resolved as FT2 because no identifiable organized or adapted-sport context was present.

During data extraction, three further pairs of duplicate records were identified that had not been detected during initial deduplication (Section 2.4). Each pair corresponded to the same publication retrieved through different database exports; in one case, the two records differed only in the order of a multilingual (Spanish, English, and Portuguese) abstract. Removing these three duplicates left 126 unique studies.

During full-text data extraction and quality appraisal, three further records were judged borderline with respect to the population or eligibility criteria and were re-evaluated by both reviewers against the locked FT1–FT7 criteria. A study of a boxing exercise program for Parkinson’s disease was excluded because it did not involve an identifiable organized or adapted-sport context (FT2). A study of sports activity after oncologic knee reconstruction was excluded because its sample did not match the target population of athletes with physical disabilities (FT1). An empirical study of a mixed-disability sample was excluded because results specific to physical disability could not be extracted separately (FT4). This left 123 unique studies.

An internal data-verification review then identified 26 potentially borderline studies for independent re-verification against the locked FT3 participation-relevance criterion. Re-verification was based on analytic linkage rather than on the mere presence of a motivational construct: studies were retained only when the factor or outcome was directly related to sport entry or initiation, continuation or persistence, withdrawal or dropout, nonparticipation, or barriers and facilitators affecting these processes. The two reviewers independently reached the same decision for 17 of the 26 records (7 agreed exclusions and 10 agreed inclusions), and these decisions were applied directly. The remaining nine records were discordant, and overall agreement for the 26 records was fair (raw agreement = 65.4%; Cohen’s κ = 0.37). Consensus review of the nine discordant records resulted in eight further exclusions and one confirmed inclusion. Together, the seven agreed exclusions and eight consensus exclusions yielded 15 FT3-based exclusions and an interim sample of 108 unique studies. To test the stability of this targeted re-verification, the motivational-factor, contextual-factor, and key-finding fields of all 108 remaining records were re-screened for the same outcome-only patterns that had prompted the 26-record audit; no additional FT3 candidates were identified. Separately, all 19 studies whose sport type or level had been recorded as not specific to a single named sport were re-examined against the locked FT2 organized or adapted-sport criterion. Seventeen were confirmed as eligible and two were excluded, yielding the final sample of 106 unique studies. The final evidence base was therefore subjected to a complete post-audit consistency screen for FT3-type outcome-only patterns and to a targeted re-check of every record with ambiguous sport specificity. No new eligibility criterion was introduced in either verification step; the existing FT criteria were re-applied. The independent reviewer files, the 26-record FT3 dispositions, the nine-record consensus record, the 19-record FT2 re-check, the duplicate-resolution log, and the final 106-study crosswalk are retained as an audit and reconciliation dataset and can be made available to the Editorial Office upon reasonable request for verification purposes.

### 2.5. Data Extraction

For each included study, data were extracted using a structured template. The template captured authors, year, country, study design (quantitative, qualitative, or mixed methods), sample characteristics (sample size, disability type, age, and sex), sport type and competitive level, participation processes or evidence categories, theoretical framework, motivational factors, contextual or environmental factors, measurement instruments, and key findings relevant to the research questions. Both reviewers (G.E.B. and U.A.) extracted data independently and verified every extracted field against the original full text, including sample size, study design, and all narrative fields (motivational factors, contextual factors, and key findings). Only data confirmed by both reviewers were retained in the final extraction dataset (Table S2). Disagreements were resolved through discussion, consistent with the consensus procedure used at the screening stages (Section 2.4).

Claude (Anthropic) was used only as a preliminary extraction aid to populate the predefined extraction fields from the content of the source documents. Both reviewers independently checked every field against the original publication and corrected or rejected any discrepancies; only human-verified information entered the final dataset. The tool was not used for eligibility decisions, participation-process coding, synthesis, or MMAT appraisal. The exact model sub-version and extraction prompt were not archived.

### 2.6. Methodological Quality Assessment

Two reviewers (G.E.B. and U.A.) independently appraised the methodological quality of the included studies using the Mixed Methods Appraisal Tool (MMAT), version 2018 [23]. The MMAT was selected because the evidence base included qualitative, randomized, non-randomized, quantitative-descriptive, and mixed-methods designs, and the tool allows all of these designs to be appraised within a single, coherent framework. Design-specific instruments, such as the Joanna Briggs Institute critical-appraisal checklists, are valid alternatives, but they would have required several separate tools for this heterogeneous corpus. The MMAT was therefore retained to ensure consistent appraisal across designs while preserving design-specific criteria. Each study was assessed against the MMAT screening questions and the five criteria for its design category, and both reviewers independently recorded criterion-level ratings (Yes, No, or Cannot tell). The complete criterion-level assessments for all 106 studies are reported in Table S4. Across 530 design-specific judgments, raw agreement was 92.3% (Cohen’s κ = 0.84), indicating almost perfect agreement [22]. Disagreements affecting 34 studies were resolved by consensus; when discussion did not fully converge, the more conservative rating was retained. For descriptive purposes only, studies were grouped as meeting ≤2, 3, or ≥4 of the five applicable criteria. These groupings are not MMAT-endorsed composite quality scores; they were used only to describe the methodological profile of the evidence and to support sensitivity analyses.

### 2.7. Data Synthesis Methods

Given the heterogeneity of study designs, populations, and measures, a narrative and thematic synthesis was conducted instead of a meta-analysis. Findings were coded and organized according to (a) the participation process or evidence category addressed and (b) two overarching categories of factors: motivational factors (e.g., intrinsic and extrinsic motivation, self-determination, and athletic identity) and contextual factors (e.g., social support, accessibility, and institutional or policy factors). Because observational and intervention designs answer different questions, intervention studies were identified and synthesized separately (Section 3.4), and their findings were not treated as equivalent to observational associations. Cross-sectional, qualitative, cohort, and other observational studies were synthesized using association, predictor, barrier, or facilitator terminology, as appropriate.

Within these two overarching categories, specific factor themes (e.g., facility accessibility, peer, coach, and team support, and cost) were developed inductively from the motivational and contextual factors extracted from each study, informed by recurring categories in the existing literature on barriers and facilitators. A study was counted toward a theme whenever its extracted factors substantively addressed that theme, regardless of whether the original authors used the same label. A single study could therefore contribute to more than one theme, and the thematic counts in Section 3.4 represent the number of studies addressing each theme rather than the number of individual mentions. Whether a factor acted as a facilitator or a barrier was recorded according to the direction reported in the original study; this information is preserved in the Motivational Factors and Contextual Factors fields of Table S2 but is not tabulated separately in the main text. Both reviewers independently performed thematic coding on the extracted factor fields for all included studies and resolved discrepancies through discussion. Thematic study counts were used only to map how often a factor appeared in the literature and were not treated as rankings of importance or as indicators of the strength of evidence. To incorporate methodological quality more transparently without converting MMAT criteria into a numerical score, the major recurring themes were also summarized by study-design mix and descriptive MMAT profile (≤2/5, 3/5, or ≥4/5 criteria met). The methodological quality profiles of the major recurring themes were also examined in relation to study-design mix. These profiles are descriptive and were not used as quantitative weights.

Participation processes and evidence categories were coded in two passes. The initial pass assigned studies to three categories: entry, maintenance or continuation, and dropout or discontinuation. After a subsequent internal data-verification review, the reviewers judged that this scheme did not adequately distinguish studies providing direct evidence of sustained participation from studies that simply described currently active participants. Explicit post hoc operational decision rules were therefore developed for Entry/Initiation, Current/General Participation, Maintenance-Related Evidence, and Withdrawal/Dropout/Nonparticipation (Table S3). Current/General Participation was treated as a cross-sectional evidence category rather than as a sequential temporal stage. To avoid equating psychological indicators of continuation with demonstrated persistence, maintenance-related studies were classified into two groups. Indirect/Prospective Maintenance-Related Evidence included intention to continue, sport commitment, future participation intentions, sustained interest, and perceived facilitators of or barriers to continuation. Observed Behavioral Persistence required evidence that participation had actually been sustained over time. Such evidence included longitudinal follow-up, repeated measurement of participation, documented retention, or clearly documented retrospective continuation. In the synthesis, retrospective evidence of persistence was distinguished descriptively from prospectively observed longitudinal persistence. The revised criteria were applied conservatively to studies previously coded as Maintenance/Persistence, and studies that only described currently active participants, without maintenance-specific evidence, were reassigned to Current/General Participation. Studies could contribute to more than one category when their design directly addressed several participation processes. Both reviewers (G.E.B. and U.A.) applied the revised framework independently; the full operational decision rules are presented in Table S3. After the 15 FT3-ineligible studies had been excluded (seven by independent agreement of both reviewers and eight through consensus resolution of nine disagreements), all participation-process and evidence-category results in Section 3 are reported for the final 106 included studies.

## 3. Results

### 3.1. Study Selection

The searches of Scopus (n = 829), Web of Science (n = 784), and PubMed/MEDLINE (n = 936) yielded 2549 records. After 900 duplicates had been removed, 1649 records were screened by title and abstract; 975 were excluded, leaving 674 records for full-text retrieval. Supplementary retrieval efforts recovered 14 journal reports that were initially unavailable. In total, 65 journal reports remained unavailable despite author contact, and 25 records were ineligible publication types (5 books, 10 book chapters, 8 conference papers, 1 web page, and 1 WHO report), leaving 584 reports for independent dual full-text assessment. Of these, 455 were excluded and 129 met the eligibility criteria after consensus. During data extraction, three duplicate pairs not detected at the identification stage were removed, leaving 126 unique studies. During quality appraisal, three further records were found to be ineligible on population or reporting grounds and were removed, leaving 123 studies. One was an exercise-intervention study in Parkinson’s disease without an identifiable sport context. A second examined post-oncologic orthopedic reconstruction outside the target population. The third was otherwise eligible, but results for physical disability could not be separated from those of a mixed-disability sample. Twenty-six studies flagged during an internal audit then underwent independent dual re-verification against the FT3 participation-relevance criterion. Both reviewers independently agreed that seven of these studies did not meet the criterion, and nine were initially discordant (raw agreement = 65.4%; Cohen’s κ = 0.37). Consensus discussion of the nine discordant records resulted in eight additional exclusions and one confirmed inclusion, giving 15 FT3-based exclusions (7 agreed and 8 by consensus) and an interim sample of 108 studies. Finally, an FT2 eligibility re-check of 19 studies with ambiguous sport specificity excluded 2 further studies, yielding the final sample of 106 studies (129 → 126 → 123 → 108 → 106; Figure 1).

### 3.2. Study Characteristics

**Figure 2:**
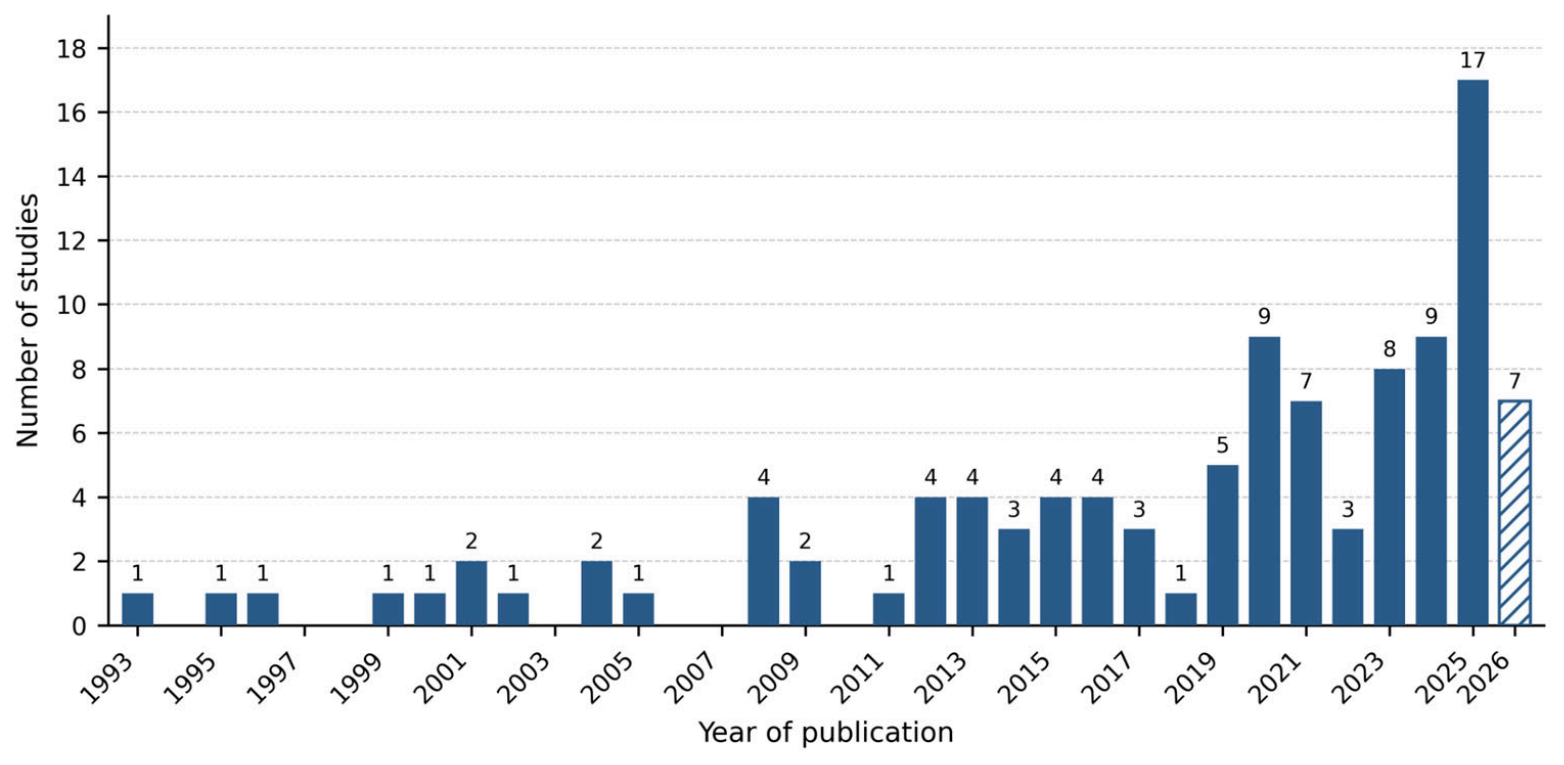
Number of included studies by year of publication (n = 106). The 2026 bar (hatched) covers a partial year, as the final searches were completed on 5 September 2026.

The 106 included studies were published between 1993 and 2026, with a marked concentration in recent years: 41 studies (38.7%) were published in 2023 or later, compared with 17 (16.0%) published before 2010 (Figure 2). All 106 included studies [11,16,17,18,19,20,21,24,25,26,27,28,29,30,31,32,33,34,35,36,37,38,39,40,41,42,43,44,45,46,47,48,49,50,51,52,53,54,55,56,57,58,59,60,61,62,63,64,65,66,67,68,69,70,71,72,73,74,75,76,77,78,79,80,81,82,83,84,85,86,87,88,89,90,91,92,93,94,95,96,97,98,99,100,101,102,103,104,105,106,107,108,109,110,111,112,113,114,115,116,117,118,119,120,121,122] are mapped individually in Table S2.

Quantitative descriptive studies were the most common design (n = 49, 46.2%), followed by qualitative studies (n = 35, 33.0%); other non-randomized quantitative designs such as cohort, secondary-data, and predictive-model studies (n = 11, 10.4%); mixed-methods studies (n = 8, 7.5%); and randomized controlled trials (n = 3, 2.8%).

The evidence base spanned at least 32 countries but was concentrated in Canada (n = 13), the United States (n = 12), the Netherlands (n = 9), Australia (n = 9), Türkiye (n = 8), and the United Kingdom (n = 7) (Table 6). Eight studies used multinational or international samples. Africa, Latin America, South Asia, and several parts of the Middle East and East Asia were comparatively underrepresented, indicating that the evidence remains concentrated in North America, Western Europe, Türkiye, and Oceania.

Sample sizes were reported for all 106 studies and ranged from 3 to 21,235 (median = 43). The upper end of this range reflects one study [24] that analyzed web-scraped International Paralympic Committee competition records rather than individually surveyed participants. Excluding this population-level record count, reported sample sizes summed to 15,004 across the remaining 105 studies; because some cohorts may overlap across reports, this figure should not be interpreted as the number of unique participants. The most frequently represented disability groups were spinal cord injury (n = 17 studies), cerebral palsy (n = 8), lower-limb amputation (n = 7), and spina bifida or myelomeningocele (n = 3). Consistent with the eligibility criteria, several further studies analyzed mixed-disability samples that included participants with visual or hearing impairments alongside a separately reported physical-disability subgroup.

**Table 5:** Summary characteristics of included studies (n = 106).

Characteristic	n (%)
**Study design**	
Quantitative descriptive	49 (46.2%)
Qualitative	35 (33.0%)
Quantitative non-randomized (cohort/predictive/secondary)	11 (10.4%)
Mixed methods	8 (7.5%)
Randomized controlled trial	3 (2.8%)
**Publication period**	
Before 2010	17 (16.0%)
2010–2019	29 (27.4%)
2020–2026	60 (56.6%)
Methodological quality (consensus MMAT criteria met, descriptive only)	
≤2/5 criteria met	45 (42.5%)
3/5 criteria met	34 (32.1%)
≥4/5 criteria met	27 (25.5%)
**Sample size (reported in 106/106 studies)**	
Median (range)	43 (3–21,235)
**Top disability populations**	
Spinal cord injury	17 (16.0%)
Cerebral palsy	8 (7.5%)
Lower-limb amputation	7 (6.6%)
Spina bifida/myelomeningocele	3 (2.8%)

**Note:** MMAT criterion counts are reported for descriptive purposes only. The MMAT does not recommend calculating or interpreting a global numerical quality score from the five design-specific criteria, and these categories were not used as quantitative study weights.

**Table 6:** Geographic distribution of included studies.

Country	k ^1^
Canada	13
USA	12
Netherlands	9
Australia	9
United Kingdom	7
Türkiye	8
Brazil	4
Chile	3
Saudi Arabia	3
Multinational/international	8
New Zealand	2
Hong Kong	2
South Korea	2
Portugal	2
Poland	2
South Africa	2
Germany	2
Other (single-study countries)	16

^1^ k = number of studies. Country assignments reflect the primary study setting; multinational or international samples are grouped separately. “Other” comprises 16 countries represented by one study each.

### 3.3. Methodological Quality (MMAT)

Two reviewers independently appraised methodological quality using the MMAT 2018 (Section 2.6), with almost perfect inter-rater agreement across the five applicable criteria (raw agreement = 92.3%; Cohen’s κ = 0.84). Criterion-level disagreements, affecting 34 of the 106 studies, were resolved by consensus, and the consensus ratings were used to assign studies to the following MMAT categories: quantitative descriptive (n = 49), qualitative (n = 35), non-randomized quantitative (n = 11), mixed methods (n = 8), and randomized controlled trial (n = 3).

For descriptive purposes only, we summarized the number of the five design-specific criteria rated “Yes” in the consensus ratings; these summaries are not an MMAT-endorsed overall quality score. Of the 106 studies, 45 (42.5%) met ≤2 of the five criteria, 34 (32.1%) met 3, and 27 (25.5%) met ≥4. The most frequent weaknesses, also reflected in the “Cannot tell” ratings, were unclear or unaddressed sample representativeness and nonresponse bias. This pattern is expected given the widespread reliance on convenience sampling at single competitions, clinics, or clubs in this literature (Section 4.1). Quality profiles for the major recurring themes are summarized, together with study-design mix, The methodological quality profiles of the major recurring themes were also examined in relation to study-design mix. These profiles are descriptive and were not used as quantitative weights.; these profiles are descriptive and are not used as quantitative weights.

### 3.4. RQ1—Motivational and Contextual Factors Associated with Sport Participation

The theoretical frameworks identified across the 106 included studies are summarized in Table 7. Of these studies, 61 did not explicitly report a theoretical framework. Self-Determination Theory and Basic Psychological Needs were represented in seven studies, followed by the Social-Ecological Model in five studies and Athletic Identity Theory in four studies. Some studies applied more than one theoretical framework; therefore, the categories are not mutually exclusive.

**Table 7:** Theoretical frameworks used in included studies (n = 106).

Theoretical Framework	k ^1^	Representative Studies
Self-Determination Theory/Basic Psychological Needs	7	[11,25,26,52,53,54,55]
Athletic Identity Theory	4	[20,27,28,29]
Social-Ecological Model	5	[30,31,32,33,34]
Health Action Process Approach (HAPA)	3	[29,35,36]
ICF (International Classification of Functioning)	3	[39,40,41]
Sport Commitment Model	1	[42]
Social Cognitive Theory	1	[38]
Social Role Valorization/Perceived Freedom in Leisure	1	[33]
Positive Psychology (grit, hardiness, resilience)	2	[43,44]
Other Named Framework (study-specific, not separately categorized above)	21	[24,40,45,46,47,48,49,50,51,62,63,66,67,71,72,78,87,100,102,110,111] (see Table S2)
No Explicit Framework Stated	61	— (see Table S2)

**Note:** Studies could contribute to more than one theoretical-framework category; percentages are therefore not presented because the categories are not mutually exclusive. ^1^ k = number of studies in which the framework was applied; some studies applied more than one framework.

#### 3.4.1. Individual/Psychological Factors

Motivational constructs drawn from Self-Determination Theory (autonomy, competence, relatedness, and intrinsic and extrinsic motivation) formed the most frequently used individual-level framework and appeared in seven studies [11,25,26,52,53,54,55]. Self-motivation and self-efficacy were identified as salient factors in eight studies [17,29,34,35,36,54,68,81], and enjoyment, fun, and flow in six studies [39,40,42,46,119,120]. Motivational constructs related to competition and competitive level were examined in only two studies [38,57]; several studies in which achievement-oriented constructs were the sole or primary focus, without a direct link to entry, continuation, or dropout, had been excluded during FT3 re-verification (Section 2.4). Athletic or disability identity was explicitly examined in five studies [20,27,28,29,49], generally as both an outcome of continued participation and a factor associated with it; one of these studies also examined how athletes’ embodied use of assistive technology shaped their identity and sense of freedom. A distinct positive-psychology strand examined grit, hardiness, and resilience as predictors of sport engagement and life satisfaction, including an original study and a later replication that added social support as a predictor [43,44].

#### 3.4.2. Interpersonal/Social Factors

Peer, coach, team, or broader social-support processes were explicitly described in 17 studies [19,44,47,49,51,55,56,60,61,63,64,65,66,67,69,79,102]. Family influences were reported in eight studies [16,35,39,45,47,68,69,71] and were most prominent at the entry stage. Examples included parental encouragement, recommendations from family members, and parental decisions that acted as gatekeepers to opportunity. Family influences were also described specifically for adolescent quad rugby athletes and for children with spina bifida or myelomeningocele. A smaller but notable group of studies highlighted the motivational role of Paralympic athletes as role models and the inspirational effect of exposure to major Games. Newer forms of technology-mediated team and peer engagement, including power-wheelchair team sport and university campus-recreation settings, were examined as emerging contexts for interpersonal sport engagement.

#### 3.4.3. Environmental/Structural Factors

Factors related to access and accessibility, including facilities, venues, infrastructure, transportation, program access, and adapted-sport opportunities, were the most frequently reported contextual factors and were identified in 21 studies [11,30,39,41,60,61,63,64,66,67,68,71,81,82,83,84,85,86,87,88,89]. Equipment and assistive-technology factors were reported in 15 studies [31,40,49,59,62,67,79,87,88,90,108,109,116,117,118]. Examples included satisfaction with sport wheelchairs, prosthesis fit and satisfaction, and access to adapted equipment. Financial, time, or broader resource burdens were reported in nine studies [11,30,68,81,82,89,92,93,94]. Institutional and policy-level factors, including federation structures, government measures, licensing bodies, and organizational support, were identified in 10 studies [30,31,68,69,83,84,93,98,99,101]. Societal attitudes, stigma, or discrimination were reported in seven studies [30,39,41,53,71,77,78], several of which addressed gendered or culturally specific barriers. One international survey identified the COVID-19 pandemic as a distinct, time-limited disruption of access to adapted sport. A large observational analysis of official competition records examined classification and eligibility structures as population-level structural correlates of Paralympic participation and the likelihood of winning a medal [24]. One qualitative study explored emerging virtual-reality training tools as a potential structural facilitator of adapted-sport skill development. Transportation or travel-access barriers were explicitly identified in only three studies [60,68,121], even though they are commonly cited in the broader literature on physical activity and disability.

Intervention evidence was limited and was interpreted separately from observational associations. Three randomized controlled trials [26,51,56] evaluated structured adapted-sport or multi-sport programs, and one further study used a non-randomized pre–post intervention design [64]. These studies provided evidence on program-related changes in motivation, activity, participation, or physical functioning; they were not combined conceptually with cross-sectional reports of factors associated with participation. One randomized trial [56] included a 3-month follow-up and thus provided the clearest intervention-based evidence in this subset that program-related gains persisted beyond the intervention period.

Quality-stratified descriptive profiles further illustrate why raw study counts should not be interpreted as rankings of evidence strength. For accessibility-related factors (k = 21), nine studies met ≤2 MMAT criteria, seven met 3, and five met ≥4. The corresponding distributions were seven, six, and four for peer, coach, team, and social support (k = 17); nine, three, and three for equipment and assistive-technology factors (k = 15); and four, four, and one for financial, time, and resource burdens (k = 9). Table 8 presents these profiles for the major recurring themes, together with study-design mix and the direction and type of evidence.

**Table 8:** Structured evidence matrix for major recurring motivational and contextual themes.

Factor/Theme	k	Design Mix	Direction/Evidence Type	MMAT Profile (Descriptive)
SDT-related motivational constructs	7	4 qualitative; 2 quantitative descriptive/cross-sectional; 1 RCT	Primarily associations/qualitative accounts; one intervention effect	≤2/5: 2; 3/5: 2; ≥4/5: 3
Self-motivation/self-efficacy	8	5 quantitative descriptive/cross-sectional; 2 qualitative; 1 quantitative analytic	Mostly positive association/predictor evidence	≤2/5: 0; 3/5: 4; ≥4/5: 4
Enjoyment/fun/flow	6	2 mixed methods; 2 quantitative descriptive/cross-sectional; 1 quantitative analytic; 1 qualitative	Mostly facilitator/association evidence	≤2/5: 4; 3/5: 2; ≥4/5: 0
Peer/coach/team/social support	17	6 qualitative; 4 mixed methods; 4 quantitative descriptive/cross-sectional; 2 RCTs; 1 pre–post intervention	Predominantly facilitator; observational and intervention evidence	≤2/5: 7; 3/5: 6; ≥4/5: 4
Family influences	8	4 quantitative descriptive/cross-sectional; 2 qualitative; 2 mixed methods	Mixed: encouragement/facilitation and gatekeeping/barrier roles	≤2/5: 4; 3/5: 2; ≥4/5: 2
Access/accessibility	21	9 qualitative; 7 quantitative descriptive/cross-sectional; 4 mixed methods; 1 pre–post intervention	Barrier when limited; facilitator when available	≤2/5: 9; 3/5: 7; ≥4/5: 5
Equipment/assistive technology	15	8 quantitative descriptive/cross-sectional; 4 qualitative; 2 mixed methods; 1 quantitative analytic	Mixed facilitator/barrier evidence	≤2/5: 9; 3/5: 3; ≥4/5: 3
Financial/time/resource burden	9	5 quantitative descriptive/cross-sectional; 3 qualitative; 1 quantitative analytic	Predominantly barrier/constraint evidence	≤2/5: 4; 3/5: 4; ≥4/5: 1
Institutional/policy factors	10	7 quantitative descriptive/cross-sectional; 3 qualitative	Mixed structural facilitator/barrier evidence	≤2/5: 6; 3/5: 4; ≥4/5: 0
Societal attitudes/stigma/discrimination	7	4 qualitative; 2 quantitative descriptive/cross-sectional; 1 mixed methods	Predominantly barrier evidence	≤2/5: 3; 3/5: 3; ≥4/5: 1

**Note:** k = number of studies addressing the theme; a single study could contribute to more than one theme. MMAT profiles are descriptive counts of studies meeting ≤2, 3, or ≥4 of the five design-specific consensus criteria; they are not MMAT-endorsed composite scores and should not be interpreted as quantitative weights.

### 3.5. RQ2—Factors Across Participation Processes and Evidence Categories

After data verification, the authors applied the revised operational criteria shown in Table S3. Entry/Initiation and Withdrawal/Dropout/Nonparticipation were retained as participation-process categories, whereas Current/General Participation was explicitly treated as a cross-sectional evidence category rather than as a sequential temporal stage. Maintenance-Related Evidence was divided into two subcategories. Indirect/Prospective Maintenance-Related Evidence comprised constructs such as intention to continue, sport commitment, sustained interest, or perceived facilitators of and barriers to continuation, without direct demonstration of continued behavior. Observed Behavioral Persistence required evidence that participation itself had been sustained over time, through longitudinal follow-up, repeated assessment of participation, or clearly documented retrospective continuation. Studies could be coded to more than one category when they directly addressed several participation processes. Contextual factors across these participation processes and evidence categories are summarized in Table 9.

**Table 9:** Contextual factors by participation process and evidence category.

Participation Process/Evidence Category	k ^1^	k MMAT ≥3/5 ^2^	Interpersonal/Environmental Factors	Representative Studies
Entry/Initiation	29	17	Family encouragement; peer/disabled-friend exposure; coach/medical-staff referral; taster/multi-sport programs; school PE exposure; facility accessibility	[17,26,32,54,56,72]
Current/General Participation	61	35	Peer/team support; coach relationships described cross-sectionally but not linked to persistence; facility/equipment accessibility; cost; institutional/policy support	[11,27,30,43,70,93]
Indirect/Prospective Maintenance-Related Evidence	8	4	Perceived need for continuous programming; club/training environment; equipment and infrastructure; economic sustainability; enabling and barrier conditions relevant to continuation	[42,46,63,65,72,89]
Observed Behavioral Persistence	5	3	School and rehabilitation environments; peer encouragement; cost and competition availability; contextual conditions documented alongside continued sport participation	[17,54,92,109,111]
Withdrawal/Dropout/Nonparticipation	11	7	Loss of or lower social support inferred from participant/nonparticipant comparisons; accessibility barriers; transportation; cost; low confidence	[35,45,68,91,106,121]

^1^ k = number of studies coded to the participation process or evidence category; studies could contribute to more than one category. ^2^ Number of studies in the category that met at least three of the five applicable MMAT criteria (sensitivity-analysis subset, n = 61; Section 3.7).

Of the 106 included studies, 61 (57.5%) were classified as Current/General Participation; this figure includes three cross-sectional studies originally labeled as Maintenance/Persistence that were reassigned because they did not provide maintenance-specific evidence. Thirteen studies (12.3%) addressed maintenance-related processes: eight (7.5%) provided indirect or prospective indicators, and five (4.7%) provided behavioral evidence of persistence. Twenty-nine studies (27.4%) addressed Entry/Initiation, and 11 (10.4%) addressed Withdrawal/Dropout/Nonparticipation. Eight studies (7.5%) contributed to more than one broad participation-process category; the category totals and percentages are therefore not mutually exclusive and exceed 106 and 100%, respectively.

The revised classification sharpens the distinction between research on currently active participants and evidence on how participation is maintained over time. More than half of the literature (57.5%) consists of single-time-point descriptions of current participants, whereas direct behavioral evidence of persistence was found in only 4.7% of the included studies, and prospective longitudinal evidence was even rarer. Study counts in this review are therefore interpreted as indicators of where research attention has been concentrated, not as estimates of the strength or causal importance of particular motivational or contextual factors. This distinction is discussed further in Section 4. The motivational factors identified across the different participation processes and evidence categories are summarized in Table 10.

**Table 10:** Motivational factors by participation process and evidence category.

Participation Process/Evidence Category	k ^1^	k MMAT ≥3/5 ^2^	Individual/Psychological Factors	Representative Studies
Entry/Initiation	29	17	Self-motivation, curiosity, prior pre-disability sport experience, risk perception/outcome expectancies (HAPA), Paralympic role-model inspiration	[17,26,32,54,56,72]
Current/General Participation	61	35	Self-Determination Theory constructs, athletic/disability identity, enjoyment/flow, grit/hardiness/resilience—measured cross-sectionally with no persistence indicator	[11,27,30,43,70,93]
Indirect/Prospective Maintenance-Related Evidence	8	4	Sport commitment, intention to continue, harmonious passion/flow, sustained interest, perceived competence, intrinsic motivation/self-determination, and motivation for continued participation	[42,46,63,65,72,89]
Observed Behavioral Persistence	5	3	Long-term sport involvement documented across developmental or participation histories; peer encouragement and self-motivation associated with continued participation; pre-/post-disability sport continuity	[17,54,92,109,111]
Withdrawal/Dropout/Nonparticipation	11	7	Amotivation, reduced self-efficacy/confidence, pain/health limitations, fear of injury, competing priorities, loss of interest or motivation	[35,45,68,91,106,121]

^1^ k = number of studies coded to the participation process or evidence category; studies could contribute to more than one category. ^2^ Number of studies in the category that met at least three of the five applicable MMAT criteria (sensitivity-analysis subset, n = 61; Section 3.7). Representative studies are selected examples cited for readability; the complete study-level mapping is provided in Table S2. Some representative studies appear in both the motivational and contextual factor summaries because individual studies could report both motivational and contextual factors.

### 3.6. RQ3—Joint Examination of Motivational and Contextual Factors

Both motivational and contextual factors were represented in the included literature, but explicit integration of the two domains within a single analytical framework was uncommon. Only five studies explicitly adopted a social-ecological model spanning several levels of influence; most focused primarily on either individual motivational constructs or contextual barriers and facilitators. The co-occurrence of motivational and contextual findings within a study should therefore not be taken as evidence that their interaction was formally tested. Overall, the literature remains divided between person-level explanations of participation and environmental or organizational accounts.

### 3.7. Methodological Profile of the Evidence Base

Cross-sectional quantitative-descriptive and qualitative interview designs predominated over randomized and longitudinal designs. Samples were typically small to moderate and frequently convenience-based, and representativeness and nonresponse bias were often insufficiently addressed. In addition, 57.5% of the studies stated no explicit guiding theoretical framework. These characteristics limit causal and predictive inference and provide important context for interpreting the thematic study counts reported above. Detailed MMAT results are presented in Section 3.3 and Table S4.

A post hoc sensitivity analysis restricted the participation-process synthesis to the 61 studies that met at least three of the five applicable MMAT criteria. The resulting pattern was materially similar to that of the full corpus of 106 studies. Current/General Participation accounted for 35/61 studies (57.4%) in the sensitivity subset versus 57.5% overall, and Entry/Initiation accounted for 17/61 (27.9%) versus 27.4% overall. Maintenance-Related Evidence accounted for 7/61 studies (11.5%) versus 12.3% overall; this comprised 4/61 (6.6%) with Indirect or Prospective indicators and 3/61 (4.9%) with Observed Behavioral Persistence. Withdrawal/Dropout/Nonparticipation accounted for 7/61 studies (11.5%) versus 10.4% overall. Thus, the predominance of cross-sectional evidence on current participation and the scarcity of direct evidence of behavioral persistence were not driven by the studies meeting fewer MMAT criteria.

## 4. Discussion

This systematic review synthesized evidence from 106 studies on motivational and contextual factors associated with sport participation among people with physical disabilities. The evidence was organized around participation processes and evidence categories covering entry and initiation, current participation, maintenance-related processes, and withdrawal, dropout, or nonparticipation. Three overarching patterns emerged.

First, consistent with the theoretical framing outlined in the Introduction, motivational and contextual factors were rarely examined in relation to each other within the same study, even though the Lancet global-perspective synthesis explicitly called for socio-ecological approaches that integrate both [1]. Only five studies explicitly adopted a social-ecological model spanning the intrapersonal, interpersonal, and structural levels simultaneously (e.g., [30,31,34]). Most examined either individual motivational constructs, most commonly those of Self-Determination Theory, or contextual barriers and facilitators, most commonly accessibility, in relative isolation. This pattern mirrors the fragmentation identified by Jaarsma et al. [6] nearly a decade ago and suggests that the field has only partially closed the integration gap they described.

Second, the revised participation-process classification reveals a marked imbalance in research attention. Most included studies (57.5%) were single-time-point descriptions of already active participants and therefore offered no direct evidence that participation was sustained over time. Although 13 studies (12.3%) addressed maintenance-related processes, eight relied on indirect or prospective indicators such as intention, commitment, sustained interest, or perceived factors of continuation, and only five (4.7%) provided behavioral evidence of persistence. Even within this small behavioral subset, prospective longitudinal observation of continued participation was uncommon. Withdrawal, dropout, or nonparticipation was addressed in 10.4% of studies, mainly through comparisons of participants and nonparticipants rather than within-person observation of discontinuation. This contrasts with the broader sport-participation literature, in which dropout has long been treated as a distinct phenomenon [8]. The Quality Parasport Participation Framework [9] identifies conditions in the physical, activity, and social environments that may support continued participation, but the evidence synthesized here rarely tested prospectively whether these conditions predict retention or dropout. The key gap is therefore not simply the small number of studies labeled as maintenance research but the scarcity of direct behavioral evidence on how participation is sustained or discontinued over time. The quality-restricted sensitivity analysis produced the same overall distribution: Current/General Participation accounted for 57.4% of the subset meeting at least three MMAT criteria, and observed behavioral persistence for only 4.9%. This descriptive pattern therefore held in the methodologically stronger subset.

Third, the methodological profile of this literature, which is predominantly cross-sectional, frequently convenience-sampled, and often atheoretical (57.5% of studies stated no guiding framework), limits causal and temporal inference. Cross-sectional associations cannot establish that a factor determines entry, produces continued participation, or causes dropout. Throughout this review, therefore, the terms association and correlate are used for observational evidence; predictor is reserved for prospective or explicitly predictive analyses; barrier and facilitator are retained when they reflect the framing of the original study; and effect and influence are reserved for intervention or otherwise causally informative evidence. Where theory was applied, Self-Determination Theory and Basic Psychological Needs Theory were the most frequently identified explicit frameworks. However, many of these studies sampled already active participants at a single time point, leaving open whether the same motivational constructs prospectively predict entry, behavioral persistence, or dropout.

The findings can also be interpreted within the International Classification of Functioning, Disability and Health (ICF) [123]. Sport participation aligns most closely with the ICF participation domain, whereas many of the contextual findings in this review, such as accessibility, transportation, equipment, social support, organizational structures, and policy, correspond to environmental factors. Motivational constructs are closer to the ICF concept of personal factors, which the ICF acknowledges but does not formally classify. This alignment offers a common language for rehabilitation and sport practitioners and underscores that participation emerges from interactions between the individual and the environment rather than from motivation alone. This interpretation applies at the level of the synthesis and does not imply that the included primary studies used the ICF framework.

Geographically, the concentration of evidence in North America, Western Europe, Türkiye, and Oceania, which together account for the large majority of included studies, leaves large regions, including sub-Saharan Africa, South Asia, and much of Latin America and the Middle East, represented by only one or two studies each. Studies from underrepresented regions, including India [68], Kenya [78], Saudi Arabia [101], and South Africa [120], reported structural and economic barriers such as scarce facilities, insufficient transformation initiatives, and limited institutional infrastructure. Such barriers were less frequently reported in the studies from higher-income countries included in this review; this should not be interpreted as evidence that they are absent in those settings. The geographic concentration of the evidence therefore limits global generalization.

### 4.1. Limitations

Several limitations should be considered when interpreting these findings. First, the search was restricted to Scopus, Web of Science, and PubMed/MEDLINE. Although these databases provide broad multidisciplinary and biomedical coverage, relevant studies indexed only in specialist, regional, or non-indexed sources, or available only as gray literature, may have been missed. SPORTDiscus, PsycINFO, CINAHL, and Embase were not searched; the executed strings did not include every plausible recreation- or maintenance-related synonym; and formal backward or forward citation chasing was not part of the original review. A post hoc sensitivity check confirmed that six clearly relevant sport-specific primary studies cited in the earlier review by Jaarsma et al. [6] had been retrieved, but this check cannot establish exhaustive retrieval. Second, the review was not prospectively registered and had no independently timestamped standalone protocol. Although the core eligibility, search, screening, extraction, and appraisal procedures were specified before screening, the participation-process framework was developed post hoc during data verification, after the initial scheme proved unable to distinguish current participation from maintenance evidence. Third, the review team comprised two authors, so disagreements were resolved by consensus between the same two reviewers rather than by an independent third adjudicator. This limitation is particularly relevant to the targeted FT3 re-verification, for which agreement was 65.4% (κ = 0.37), although all final decisions were documented and the remaining evidence base was subsequently rechecked against the locked criteria. Fourth, two included studies were assessed partly on the basis of translated summaries rather than a full reading in the original language [70,106], which may have reduced interpretive precision. Fifth, substantial heterogeneity in study designs, populations, sports, and outcomes precluded meta-analysis; thematic study counts therefore describe patterns of research attention rather than pooled effect sizes or strength of evidence. Sixth, participation-process coding required reviewer interpretation because most primary studies did not use the categories employed in this review. The revised framework distinguishes cross-sectional evidence on current participation, indirect or prospective maintenance indicators, and observed behavioral persistence, and it should not be interpreted as a set of strictly sequential temporal stages. Seventh, 42.5% of the included studies met no more than two of the five applicable MMAT criteria. Frequency counts were therefore not treated as rankings of evidence strength, and findings from studies with greater methodological limitations were interpreted cautiously. Eighth, the extensive verification and reconciliation process revealed weaknesses in the stability and documentation of some initial screening decisions. Although the final decisions were rechecked against the locked eligibility criteria and are supported by retained audit records, residual error cannot be ruled out. Finally, no formal assessment of reporting bias or certainty of evidence (e.g., GRADE) was undertaken. The frequency with which a factor appears in the included literature may therefore reflect not only its substantive relevance but also research priorities, measurement choices, publication practices, and selective reporting, and thematic frequencies should not be interpreted as estimates of the certainty of evidence.

### 4.2. Future Research

Four priorities for future research emerge from these findings. First, studies should directly observe behavioral persistence and withdrawal over time rather than inferring maintenance from intention, commitment, or the characteristics of currently active participants. Prospective cohort studies with repeated measurement of participation would be particularly valuable for identifying when and why continuation or dropout occurs. Second, motivational and contextual factors should be examined jointly in integrated social-ecological or multilevel designs, so that psychological motivation can be studied in relation to access, cost, transportation, coaching, equipment, and organizational opportunity. Third, research should be extended to regions underrepresented in the included literature, particularly sub-Saharan Africa, South Asia, and Latin America. Such geographic diversification is needed to test whether the balance and interaction of structural and motivational factors vary across economic, cultural, and service contexts. Fourth, more theory-driven research is required. Self-Determination Theory and the Quality Parasport Participation Framework provide useful starting points, but prospective studies should test whether their constructs predict entry, continued participation, and dropout, rather than assuming that associations observed among current participants generalize across participation processes.

## 5. Conclusions

This systematic review synthesized 106 studies on motivational and contextual factors associated with sport participation among people with physical disabilities. The evidence was dominated by cross-sectional descriptions of currently active participants (57.5%); maintenance-related processes were addressed in 12.3% of studies, and behavioral evidence of persistence was found in only 4.7%. Entry or initiation was addressed in 27.4% of studies and withdrawal, dropout, or nonparticipation in 10.4%, with evidence on dropout relying mainly on comparisons of participants and nonparticipants rather than within-person observation over time. Motivational and contextual factors were both prominent but were seldom examined within genuinely integrated analytical designs, and more than half of the studies stated no explicit theoretical framework. For researchers, the priority is prospective, theory-driven studies that measure continuation and discontinuation directly and integrate motivational and structural conditions. For practitioners and sport organizations, the findings indicate that efforts to support participation should not focus on individual motivation alone: access, social support, cost, equipment, coaching, and organizational conditions must be addressed alongside psychological factors. Overall, the literature is considerably better at describing people who already participate than at explaining, with direct temporal evidence, how sport participation is initiated, sustained, or discontinued.

## Data Availability

No new primary participant-level data were collected in this study. The systematic review was based on data extracted and synthesized from previously published studies. Supporting information, including the PRISMA 2020 checklist, study characteristics, operational coding rules, and detailed methodological quality appraisals, is provided in Supplementary Tables S1–S4. Additional audit and reconciliation records are retained by the authors and may be made available to the Editorial Office upon reasonable request for verification purposes.

## Supplementary Materials

The following supporting information can be downloaded at: https://www.mdpi.com/article/10.3390/sports14100457/s1, Table S1: PRISMA 2020 checklist [15]; Table S2: Characteristics of included studies; Table S3: Operational decision rules for participation-process and maintenance-evidence coding; Table S4: Detailed MMAT quality appraisal of included studies.
