# Supplementary material for: Motivational and Contextual Factors Across Sport Entry, Current Participation, Maintenance, and Withdrawal Among People with Physical Disabilities: A Systematic Review
Source: Sports (Basel). 2026 Oct 14;14(10):457. doi: 10.3390/sports14100457 (PMC13709342; doi:10.3390/sports14100457)
Supplement: Supplementary file 1 [file sports-14-00457-s001.zip › Table_S1_PRISMA_Checklist.pdf]

Table S1. PRISMA 2020 checklist

Motivational and Contextual Factors Across Sport Entry, Current Participation, Maintenance, and Withdrawal Among People with Physical Disabilities: A Systematic Review

Section/Topic	Item #	Checklist Item	Location Where Item Is Reported
**TITLE**			
Title	1	Identify the report as a systematic review.	Title page
**ABSTRACT**			
Abstract	2	See the PRISMA 2020 for Abstracts checklist.	Abstract
**INTRODUCTION**			
Rationale	3	Describe the rationale for the review in the context of existing knowledge.	Section 1, 1.2
Objectives	4	Provide an explicit statement of the objective(s) or question(s) the review addresses.	Section 1.3 (RQ1–RQ4)
**METHODS**			
Eligibility criteria	5	Specify the inclusion and exclusion criteria for the review and how studies were grouped for the syntheses.	Section 2.2, Table 1 (PICOS)
Information sources	6	Specify all databases and other sources searched, and the date each was last searched.	Section 2.3
Search strategy	7	Present the full search strategies for all databases, including filters and limits used.	Section 2.3, Table 2
Selection process	8	Specify the methods used to decide whether a study met the inclusion criteria, including number of reviewers and independence.	Section 2.4
Data collection process	9	Specify methods used to collect data from reports, including number of reviewers and independence.	Section 2.5
Data items	10a/10b	List and define all outcomes and other variables for which data were sought.	Section 2.5
Risk of bias assessment	11	Specify methods used to assess risk of bias, including tool(s) used and reviewer independence.	Section 2.6 (MMAT)
Effect measures	12	Specify effect measure(s) used in synthesis. Not applicable—narrative/thematic synthesis; no pooled effect measures were calculated.	Section 2.7
Synthesis methods	13a–13f	Describe methods used to synthesize results and the rationale for the choice.	Section 2.7, Sections 3.4–3.6
Reporting bias assessment	14	Describe methods used to assess risk of bias due to missing results.	Not formally assessed—see Limitations (4.1)
Certainty assessment	15	Describe methods used to assess certainty of evidence for an outcome.	Not formally assessed (e.g., GRADE)—see Limitations (4.1)
**RESULTS**			
Study selection	16a/16b	Describe results of search and selection, ideally with a flow diagram.	Section 3.1, Figure 1
Study characteristics	17	Cite each included study and present its characteristics.	Section 3.2, Table 5, Table S2
Risk of bias in studies	18	Present assessments of risk of bias for each included study.	Section 3.3; Table S4
Results of individual studies	19	Present, for each study, summary statistics and characteristics.	Table S2
Results of syntheses	20a–20d	Summarize and present results of all syntheses conducted.	Sections 3.4–3.6, Tables 7–9
Reporting biases	21	Present assessment of risk of bias due to missing results for each synthesis.	Not formally assessed—see Limitations (4.1)
Certainty of evidence	22	Present assessment of certainty of evidence for each outcome.	Not formally assessed—see Limitations (4.1)
**DISCUSSION**			
Discussion	23a	Provide general interpretation of results in context of other evidence.	Section 4
	23b	Discuss limitations of the evidence included in the review.	Section 4.1
	23c	Discuss limitations of the review processes used.	Section 4.1
	23d	Discuss implications for practice, policy, and future research.	Section 4.2

Section/Topic	Item #	Checklist Item	Location Where Item Is Reported
**OTHER INFORMATION**			
Registration and protocol	24a–24c	Provide registration information, or state review was not registered; indicate protocol availability.	Section 2.1—not registered in a public registry (stated)
Support	25	Describe sources of financial or non-financial support and role of funders.	Funding section
Competing interests	26	Declare any competing interests of review authors.	Conflicts of Interest section
Availability of data, code and other materials	27	Report which materials/data/code are publicly available and where they can be found.	Data Availability Statement; Supplementary Materials

From: Page MJ, McKenzie JE, Bossuyt PM, Boutron I, Hoffmann TC, Mulrow CD, et al. The PRISMA 2020 statement: an updated guideline for reporting systematic reviews. BMJ 2021;372:n71. doi: 10.1136/bmj.n71.
