# Supplementary material for: Motivational and Contextual Factors Across Sport Entry, Current Participation, Maintenance, and Withdrawal Among People with Physical Disabilities: A Systematic Review
Source: Sports (Basel). 2026 Oct 14;14(10):457. doi: 10.3390/sports14100457 (PMC13709342; doi:10.3390/sports14100457)
Supplement: Supplementary file 1 [file sports-14-00457-s001.zip › Table_S2_Study_Characteristics.pdf]

Table S2. Characteristics of included studies (n = 106)

Motivational and Contextual Factors Across Sport Entry, Current Participation, Maintenance, and Withdrawal Among People with Physical Disabilities: A Systematic Review

No.	Author(s), Year	Country	Study Design	Sample Size (N)	Disability Type	Age Range	% Female	Sport Type/Level	Participation Process / Evidence Category	Theoretical Framework	Motivational Factors	Contextual Factors	Measurement Instruments	Key Findings	MMAT Category	Consensus MMAT Criteria Met (descriptive only)
14	McLoughlin, Weisman Fecske, Castaneda, Gwin & Graber, 2017	USA	Qualitative	23	Physical disability (elite athletes)	mean 24.3	26%	Multiple elite/adapted sports	Current/General Participation	Self-Determination Theory	Intrinsic/autonomous motivation constructs (SDT); overcoming barriers as a motivational driver	Cost; time constraints; lack of opportunity (barriers); empowerment/advocacy; college scholarships; performance-related goals (facilitators)	Semi-structured interview guide; multi-researcher thematic coding	Six themes emerged: athletes attributed participation to SDT constructs and to overcoming barriers (cost, time, opportunity); facilitators to athletic development included empowerment/advocacy, increased health, college scholarships, and achieving performance goals	1 Qualitative	≥4/5
18	Severin, Kinderen & Baumgart, 2025	International	Quantitative (secondary/web-scraped data analysis)	21235	Physical disability (elite Paralympic athletes, multiple impairment types)	N/R	N/R	Multiple (all Paralympic sports)	Current/General Participation	IPC eligible-impairment classification framework (10 impairment types)	Not the primary focus (structural/classification factors are the focus)	Athlete origin (acquired vs. congenital impairment) and impairment type examined as structural determinants of participation and medal-winning likelihood across different Paralympic sports	Competition data and athlete biographies web-scraped from the International Paralympic Committee website (www.Paralympic.org)	Analyzed whether athlete origin (acquired or congenital impairment) and impairment type affect participation levels and likelihood of winning a medal across different Paralympic sports, using web-scraped IPC competition data and athlete biographies; found some sports show overrepresentation of certain origin/impairment groups	3 Non-randomized	≤2/5
19	Yilmaz, Kirimoglu & Mirze, 2020	Turkey	Quantitative descriptive	41	Physical disability (n=24, wheelchair basketball) + hearing impairment (n=17, football)—physical-disability subgroup results reported separately	12–60	0% female reported for physically-disabled subgroup (not specified separately, sample described as volunteer athletes)	Wheelchair basketball (professional league)	Current/General Participation	Self-Determination Theory (Deci & Ryan, 1985)	Intrinsic motivation; extrinsic motivation; amotivation, measured via Sports Participation Motivation Scale for Disabled Individuals (SPMSDI)	Disability type and sport branch/competitive level associated with motivation differences (physically-disabled wheelchair-basketball players—professional league—scored higher on intrinsic/extrinsic motivation than hearing-impaired amateur football players); age and educational status not significant	Sports Participation Motivation Scale for Disabled Individuals (SPMSDI; Tekkurşun et al., 2018); Personal Information Form	Physically disabled athletes (professional wheelchair basketball) showed significantly higher intrinsic and extrinsic motivation than hearing-impaired athletes (amateur football); no significant amotivation difference; age/education not associated with motivation; Note: disability type is confounded with competitive level (professional vs. amateur) and sport branch in this sample	4 Quantitative descriptive	≤2/5
20	Ben Rakaa, Lourenço, Bassiri & Lotfi, 2025	Morocco	RCT	96	Motor disability (school-age para-athletics classes 30/40/50)	mean 16.79 (SD 0.87)	N/R	School para-athletics (adapted PE + competitions)	Entry/Initiation	Self-Determination Theory (SMS-28 Sport Motivation Scale)	Intrinsic motivation to stimulation (+205% in combined APE+Para-Athletics group)	Adapted Physical Education (APE) program; combined APE + para-athletics training/competition (24 sessions, 3 months) vs. no-intervention control	SMS-28 (Sport Motivation Scale); Ryff's Psychological Well-Being Scale; pre/post design	The APE+Para-Athletics group showed marked improvements across nearly all motivation domains (+205% intrinsic motivation to stimulation) and psychological well-being (autonomy, positive relationships); APE-only group improved to a lesser extent; control group showed minimal change	2 Randomized controlled trial	3/5
21	Tasiemski & Brewer, 2011	Poland/USA (SCI sample nationality not specified in excerpt)	Quantitative descriptive	1034	Spinal cord injury	N/R	N/R	Multiple (team vs. individual sports compared)	Current/General Participation	Athletic Identity theory	Athletic identity (positively related to current weekly sport participation, controlling for age/gender/pre-SCI participation)	Ability to practice one's favorite sport after SCI (associated with higher athletic identity and better psychological adjustment); team vs. individual sport type	Athletic identity measure; life satisfaction, anxiety, and depression measures	Current weekly sport participation was positively related to athletic identity; being able to continue one's favorite sport after SCI was associated with higher athletic identity and better psychological adjustment; team-sport participants reported better psychological adjustment than individual-sport participants	4 Quantitative descriptive	≥4/5
22	Tasiemski, Kennedy, Gardner & Blaikley, 2004	United Kingdom (Poland-based lead author)	Quantitative descriptive	678	Spinal cord injury	N/R	N/R	Multiple (athlete and non-athlete SCI compared)	Current/General Participation	Athletic Identity theory	Athletic identity (AIMS scores varied by gender, athlete status, and weekly sport-participation hours; lower overall than able-bodied/other-disabled comparators)	Reasons for and barriers to sports participation examined (specific barriers)	Athletic Identity Measurement Scale (AIMS, 7-item version psychometrically evaluated)	Among 678 people with SCI, athletic identity (AIMS) was lower than in able-bodied adults/adolescents and other physically disabled comparators, and varied by gender, athlete status, and participation hours; athletic identity was not related to depression, anxiety, or life satisfaction; exploratory factor	4 Quantitative descriptive	3/5

No.	Author(s), Year	Country	Study Design	Sample Size (N)	Disability Type	Age Range	% Female	Sport Type/Level	Participation Process / Evidence Category	Theoretical Framework	Motivational Factors	Contextual Factors	Measurement Instruments	Key Findings	MMAT Category	Consensus MMAT Criteria Met (descriptive only)
														analysis did not fully support the original AIMS structure in this population		
23	Maciá-Andreu, Vaquero-Cristóbal, Meroño, Abenza-Cano, García-Roca, Cánovas-Álvarez, Díaz-Aroca, Capranica, Stanescu, Pereira, Doupona, Mendes, Figueiredo, Isidori, Sánchez-Pato & Leiva-Arcas, 2023	Spain (multi-European sample)	Quantitative descriptive (cross-national survey)	203	Physical disability (student-athletes)	N/R	N/R	Multiple (student-athletes, varying professionalization levels)	Current/General Participation	Dual Career framework; Athletic Identity	Athletic identity (analyzed by disability type and professionalization level)	Dual-career barriers (balancing sport and education)—perceived barriers to dual-career success		Analyzed perceived barriers to dual-career success and athletic identity among 203 student-athletes with disabilities from five European countries, according to disability type and level of professionalization	4 Quantitative descriptive	≤2/5
24	Perrier, Sweet, Strachan & Latimer-Cheung, 2012	Canada	Quantitative (structural/predictive model)	101	Acquired physical disability	mean 44.0 years (SD 12.8)	81.2% (82/101)	Sport (general)	Current/General Participation	Health Action Process Approach (HAPA); Athletic Identity	Athletic identity and HAPA constructs (self-efficacy, planning, etc.) jointly predicting sport participation		HAPA-based questionnaire; Athletic Identity measure	Tested the fit of the Health Action Process Approach (HAPA) model, extended with athletic identity, for predicting sport participation among individuals with acquired physical disabilities	3 Non-randomized	3/5
25	Mulligan, Miyahara & Nichols-Dunsmuir, 2017	New Zealand	Qualitative (multi-stakeholder)	53	Long-term mobility impairment (plus service providers/funders as informants)	N/R	N/R	Physical activity (general, not sport-specific)	Current/General Participation	Social-Ecological Model (4 levels)	Not the primary focus (study centers on structural/systemic barriers)	Physical and financial circumstances, social attitudes, built environment (individual level); fiscal/organizational constraints (service-provider level); funding/planning decisions removing accessibility features to save money (policy level)	Interviews (people with mobility impairment, service providers, service planners/funders across 5 NZ locations); thematic analysis mapped to social-ecological framework levels	Interviewed 53 participants (people with mobility impairment, service providers, and planners/funders) across New Zealand, finding that barriers are sustained by financial, administrative, and attitudinal constraints across all social-ecological levels, including funders removing planned accessibility features to cut costs	1 Qualitative	3/5
26	Castelli Correia de Campos, Fica, Fernandéz, Teixeira Fabricio dos Santos, Gamonales, Winckler, Pena, Muñoz, Pleticosic, Luarte Rocha, Rossato de Victo, del Val Martín & Ferrari, 2025	Chile	Quantitative descriptive	35	Physical disability (wheelchair users and non-wheelchair users)	mean 26.0 years (SD 10.5)	N/R	Multiple (varied sport practice types)	Current/General Participation	Social-Ecological Model	Not the primary focus (study centers on structural/organizational barriers)	Organizational level was the main barrier (lack of adapted outdoor spaces; lack of information/inclusive advertising in sports centers); differences observed between wheelchair-user and non-wheelchair-user groups; community context also salient	Physical Activity Questionnaire for People with Mobility Impairments (BPAQ-MI), assessing 4 social-ecological levels	Organizational and community-context barriers were the main perceived barriers to physical activity among Chilean athletes with disability, with differences by disability/wheelchair-use type	4 Quantitative descriptive	≤2/5
27	Leung, Chung & Chu, 2020	Hong Kong (China)	Qualitative	20	Physical impairment (adults)	mean 53.52 (SD 9.02)	60%	Sitting light volleyball (SLVB)	Entry/Initiation	Social-Ecological Model	Psychological attributes/experiences of intervention participation	16-week structured intervention format; suitability/feasibility of SLVB as a new adaptive sport	Semi-structured interviews	Examined participants' experiences of a 16-week sitting light volleyball intervention for adults with physical impairments in Hong Kong, assessing suitability and feasibility of SLVB as a physical activity/sport using a social-ecological lens	1 Qualitative	≥4/5
28	Aytur, Craig, Frye, Bonica, Rainer, Hapke & McGilvray, 2018	USA	Qualitative (Photovoice)	8	Physical disability (youth sled hockey athletes)	9–16 years (mean 13.6)	12.5% (1/8)	Sled hockey (competitive, community-based adaptive sport)	Current/General Participation	Social Role Valorization; Perceived Freedom in Leisure; Socio-Ecological Model	Identification with valued social roles; perceived freedom in leisure	Adaptive sport programs as 'bridging organizations' connecting individual capacities to valued social roles; barriers and enablers of participation examined via socio-ecological levels	Photovoice (template analytic method)	Competitive sport participation (1) facilitated identification with valued social roles, (2) supported Perceived-Freedom-in-Leisure outcomes, and (3) empowered youth with disabilities to articulate barriers/enablers to social inclusion; photovoice is highlighted as a tool for	1 Qualitative	3/5

No.	Author(s), Year	Country	Study Design	Sample Size (N)	Disability Type	Age Range	% Female	Sport Type/Level	Participation Process / Evidence Category	Theoretical Framework	Motivational Factors	Contextual Factors	Measurement Instruments	Key Findings	MMAT Category	Consensus MMAT Criteria Met (descriptive only)
														informing therapeutic recreation and adaptive sport practice		
29	Epiney, Hany, Nagel & Klenk, 2026	Switzerland	Quantitative descriptive (explorative cross-sectional)	259	Physical disability (Swiss Paraplegic Association members)	N/R	N/R	Wheelchair sports (organized club-based groups)	Current/General Participation	Social-Ecological Model	Well-being (WHO-5 Well-Being Index); exercise self-efficacy	Club-related characteristics (individual, interpersonal, and club-level factors examined jointly)	Online questionnaire; WHO-5 Well-Being Index; Exercise Self-Efficacy Scale	Applied a social-ecological model to investigate individual, interpersonal, and club-related factors of physical activity participation among 259 members of Swiss wheelchair sport groups (Swiss Paraplegic Association), examining well-being and self-efficacy as individual-level predictors	4 Quantitative descriptive	≥4/5
30	Shirazipour, Latimer-Cheung & Arbour-Nicitopoulos, 2015	Canada	Qualitative	20 parents (10 athletes; 10 non-athletes)	Mobility impairment (youth; parents of athletes AND non-athletes)	children 7–16 years; parent mean age 42.5/42.2	N/R	Sport (general, parental support decisions)	Entry/Initiation, Withdrawal/Dropout/Nonparticipation	Health Action Process Approach (HAPA)—applied to parents	Parental self-efficacy (multiple types); planning constructs	Parental decision-making and commitment as a structural/social determinant of youth sport participation (three-fold increase in participation linked to parental commitment, per cited literature)	Qualitative interviews (exploratory)	Exploratory qualitative investigation of HAPA's relevance for understanding parental decisions to support sport participation for youth with mobility impairment; novel inclusion of parents of BOTH athletes and non-athletes	1 Qualitative	≥4/5
31	Perrier, Shirazipour & Latimer-Cheung, 2015	Canada	Quantitative descriptive (group comparison)	201	Acquired physical disability	N/R	N/R	Adapted sport (general)	Entry/Initiation	Health Action Process Approach (HAPA)	Risk perception; outcome expectancies; task self-efficacy (HAPA constructs)—hypothesized to differ across non-intenders, intenders, and actors	Demographic and disability-related group differences also examined	HAPA-based questionnaire constructs	Compared non-intenders, intenders, and actors (sport participation stages) on demographic, disability, and HAPA constructs; hypothesized non-intenders would score lowest and actors highest on all HAPA constructs, with intenders differing from both	4 Quantitative descriptive	≥4/5
32	Perrier, Latimer-Cheung, Martin Ginis & SHAPE-SCI Research Team, 2012	Canada	Quantitative (secondary cross-sectional analysis of a cohort)	695	Spinal cord injury	N/R	N/R	Sport and exercise within leisure-time physical activity (general)	Current/General Participation	None stated explicitly (social-cognitive predictors measured)	Social-cognitive predictors of moderate-to-vigorous leisure-time physical activity (MV-LTPA)	Seasonal variation (community setting, Ontario, Canada) as an environmental/temporal context of activity and sport participation	Telephone interviews; logistic regression for participation; hierarchical linear regression in the active subcohort (n = 342)	Among 695 people with spinal cord injury, season did not predict whether participants engaged in sport; winter was associated with lower total MV-LTPA and exercise, but not sport participation	3 Non-randomized	3/5
33	Stapleton, Perrier, Campbell, Tawse & Martin Ginis, 2016	Canada	Quantitative (predictive model)	95	Physical disability (parasport competitors)	mean 34.36 years (SD 12.41)	26%	Multiple (wheelchair sports, competitive levels compared)	Current/General Participation	Social Cognitive Theory	Modifiable social-cognitive factors predicting higher vs. lower parasport competitive level	Not the primary focus (individual-level social-cognitive predictors are the focus)	Social Cognitive Theory-based questionnaire battery	Tested a Social Cognitive Theory model to predict participation in higher versus lower levels of parasport competition, identifying modifiable factors associated with reaching higher competitive levels	3 Non-randomized	≤2/5
34	Jaarsma, Dijkstra, de Blécourt, Geertzen & Dekker, 2015	Netherlands	Mixed methods	30	Physical disability (67% cerebral palsy); children	mean 14.1 (SD 2.9)	42%	Multiple (swimming, cycling, football most common)	Entry/Initiation	International Classification of Functioning (ICF) framework referenced (diagram)	Dependency on others (child-reported barrier); fun and social contacts (facilitators)	Parents' lack of information about sports facilities; family attitude influence (professional-reported); health benefits (facilitator)	Questionnaire (children and parents, n=30/38); semi-structured interviews (17 professionals); triangulated mixed-method design	77% of children participated in sports after school; children, parents, and professionals reported different barriers (dependency on others; lack of information; family attitude) and shared facilitators (health benefits, fun, social contacts); sports participation of children with physical disabilities is a complex, multi-perspective phenomenon	5 Mixed methods	≤2/5
35	Jaarsma, Dekker, Geertzen & Dijkstra, 2016	Netherlands	Quantitative descriptive (comparative survey)	1223	Physical disability (post-rehabilitation)	mean 51.6 years (SD 15.1)	N/R	Sports (strictly defined, excluding general physical activity)	Withdrawal/Dropout/Nonparticipation	Theory of Planned Behavior; International Classification of Functioning (ICF)	Health, fun, increasing physical strength, younger age, and higher education were positively associated with sport participation; advice from rehabilitation professionals was a facilitator	Use of assistive devices and environmental barriers were negatively associated with sport participation; inactive and active participants were compared on experienced barriers and facilitators	Self-constructed questionnaire on sport participation, barriers, and facilitators; comparative analysis of inactive versus active participants	Among 1,223 adults after rehabilitation, 58% were active in sport; younger age and higher education were positively associated with participation, whereas assistive-device use and environmental barriers were negatively associated; health, fun, strength, and professional advice were key facilitators	4 Quantitative descriptive	3/5
36	Nam, Lee, Jun, Parke, Kim & Chae, 2016	South Korea	Quantitative descriptive (survey)	62	Spinal cord injury (physically active)	mean 42.8 years (SD 13.5)	14.5% (9/62)	Sports (general, ICF-based)	Current/General Participation	International Classification of Functioning, Disability and Health (ICF)—ICF Core Set for SCI	Not the primary focus (study centers on structural/systemic barriers)	Individual- and societal-level barriers per ICF Core Set (112 categories); body function/structure barriers (pain, urination, defecation, muscle tone, joint mobility,	ICF Core Set for SCI-based questionnaire (112 categories, 32 second-level body-function/structure categories); unmet medical care assessment	Explored dilemmas of Korean athletes with SCI participating in sports, identifying facilitators and barriers using the ICF framework; found systemic/environmental	4 Quantitative descriptive	≤2/5

No.	Author(s), Year	Country	Study Design	Sample Size (N)	Disability Type	Age Range	% Female	Sport Type/Level	Participation Process / Evidence Category	Theoretical Framework	Motivational Factors	Contextual Factors	Measurement Instruments	Key Findings	MMAT Category	Consensus MMAT Criteria Met (descriptive only)
												skin protection); systemic/environmental support identified as lower than needs (e.g., inadequate nearby facilities)		support was lower than needs, with common barriers including pain, urination/defecation issues, muscle tone, joint mobility, and skin protection		
37	Yao & Shapiro, 2024	USA (likely; not confirmed in excerpt)	Quantitative (model-testing/SEM)	157	Physical disability	18–68 years (mean 34.87, SD 11.78)	N/R	Multiple (not sport-specific in abstract)	Indirect/Prospective Maintenance-Related Evidence	Sport Commitment Model (Scanlan et al.)	Enjoyment; personal investment; involvement opportunities; involvement alternatives	Social constraints	Sport Commitment Model questionnaire battery	Examined the Sport Commitment Model in athletes with disabilities: magnitude of antecedent factors' (enjoyment, personal investment, involvement opportunities, social constraints, involvement alternatives) contribution to commitment, and compared original SCM vs. mediation vs. direct/indirect model structures	3 Non-randomized	≤2/5
38	Martin, Byrd, Watts & Dent, 2015	USA	Quantitative (predictive/regression)	75	Physical disability (wheelchair basketball athletes)	N/R	1%	Wheelchair basketball	Current/General Participation	Positive psychology constructs (grit, hardiness, resilience)	Grit, hardiness, and resilience predicting sport engagement (22% variance) and general/sport-specific life satisfaction (26% variance)	Not the primary focus (individual psychological traits are the focus)	Grit scale; hardiness measure; resilience measure; life satisfaction and sport engagement measures	Among 75 wheelchair basketball athletes (74 men, 1 woman), hardiness and resilience significantly predicted life satisfaction (26% variance explained), while resilience and grit significantly predicted sport engagement (22% variance explained); athletes highest in grit and resilience were the most engaged	4 Quantitative descriptive	3/5
39	Atkinson & Martin, 2020	USA	Quantitative (correlational/regression)	87	Physical disability (disability sport context)	19–61 years (mean 35.94, SD 9.26)	8.0% (7/87)	Disability sport (general/multiple)	Current/General Participation	Positive psychology constructs (grit, hardiness, resilience)	Grit; hardiness; resilience—predicting sport engagement and life satisfaction	Athletic social support (added predictor extending prior model)	Grit scale; hardiness measure; resilience measure; athletic social support measure; life satisfaction measure	Replication and extension of Martin, Byrd, Watts & Dent (2015): grit, hardiness, and resilience predicted sport engagement and life satisfaction; athletic social support was examined as an additional predictor	3 Non-randomized	3/5
40	Buffart, van der Ploeg, Bauman, van Asbeck, Stam, Roebroeck & van den Berg-Emons, 2008	Netherlands	Quantitative descriptive (cross-sectional)	51	Myelomeningocele (spina bifida)	mean 21.1 (SD 4.5)	49%	General/unspecified organized sport (not sport-specific)	Withdrawal/Dropout/Nonparticipation	None explicitly named; draws on perceived-competence/self-worth constructs (Harter-type self-perception framework implied)	Perceived athletic competence; physical appearance; global self-worth (trend, p=0.10); personal preference emphasized over physical ability in conclusion	Social support from family (significant correlate); disease-related characteristics (ambulatory status, hydrocephalus) NOT associated with participation	Self-report questionnaires (social support, perceived competence, exercise enjoyment); objective physical activity monitor (accelerometry); peak oxygen uptake test; muscle strength and body-fat measurement	69% (35/51) participated in sports; sports participation was associated with family social support, perceived athletic competence, and physical appearance (p<=0.05), not with disease-related characteristics; sports participants had higher self-reported (but not objectively measured) physical activity and non-significantly better muscle strength/peak oxygen uptake; authors conclude participation reflects personal preference rather than physical ability	4 Quantitative descriptive	3/5
41	Bogado & Villalba, 2023	Argentina	Mixed methods	31	Motor disability (federated athletes)	N/R	N/R	Multiple parasports (high-performance/federated)	Indirect/Prospective Maintenance-Related Evidence	Dualistic Model of Passion (Vallerand); Flow Theory	Harmonious passion; frequent/deep flow states; perceived sport competence (moderately high)	Favorable training/development environments (general, not itemized in abstract)	Perceived Competence questionnaire; Passion Scale (Vallerand et al., 2003); Flow short scale (Fernández et al., 2015); Future Practice Intention Scale; 6 semi-structured interviews	Athletes reported harmonious passion, frequent/deep flow, moderately high perceived competence; intention to continue practicing and competing was very high; authors recommend interventions fostering harmonious passion and positive experiences to support permanence in sport	5 Mixed methods	≤2/5
42	Clutterbuck, Auld & Johnston, 2022	Australia	Mixed methods	39	Cerebral palsy (ambulant, school-aged; GMFCS I=11, II=28)	6-12 years	31%	Multi-sport peer-group intervention (Sports Stars)	Entry/Initiation	Physical Literacy framework	Sports activity competence across physical, social, cognitive, and psychological physical-literacy domains	Community-based peer-group intervention design preferred by physiotherapists (8/8) and parents (26/29); practitioner-led delivery	Session Reports; Fidelity Evaluations (28 children); Perspectives Surveys (29 parents, 8 physiotherapists)	Over 84% of children attended most sessions; physiotherapists and parents reported improvements across physical, social, cognitive and psychological domains; a community-based peer-group format was the preferred intervention design for sports-focused goals; parents/physiotherapists	5 Mixed methods	≤2/5

No.	Author(s), Year	Country	Study Design	Sample Size (N)	Disability Type	Age Range	% Female	Sport Type/Level	Participation Process / Evidence Category	Theoretical Framework	Motivational Factors	Contextual Factors	Measurement Instruments	Key Findings	MMAT Category	Consensus MMAT Criteria Met (descriptive only)
														perceived Sports Stars as effective and acceptable		
43	Blinde & McCallister, 1999	USA	Qualitative (sociological analysis)	16	Physical disability (women)	19–54 years (mean 31.5)	100%	Sport and physical fitness activity (general)	Entry/Initiation	Gender/feminist sport sociology framework (intersectionality of gender and disability)		Gendered social dynamics of sport entry: masculine hegemony in sport; presumed physical differences between men/women; images of male domination/superiority as structural/cultural barriers		Examined the intersection of gender and disability dynamics in women's entry into and experiences of sport and physical fitness activity, situating findings within broader sport-sociology literature on masculine hegemony and gender dynamics in sport	1 Qualitative	≤2/5
44	Vila-Nova, Santos, João & Pascoal, 2026	Portugal	Qualitative	8	Cerebral palsy (GMFCS II-IV)	9-16 years	N/R	Frame football (adapted)	Current/General Participation	F-words for Child Development framework (Function, Family, Fitness, Fun, Friends, Future)	Claiming identity as a football player (confidence, pride via embodied participation)	Belonging through shared experience (social connection, emotional safety with teammates); assistive devices reimagined as enabling tools (adapted play)	Semi-structured interviews (8 children + 10 parents); Framework Method analysis	Three themes: (1) Redefining Ability through Adapted Play, (2) Belonging through Shared Experience, and (3) Claiming Identity as a Football Player; children embraced athletic roles, building confidence and pride through frame football participation	1 Qualitative	≥4/5
45	Day, 2013	United Kingdom	Qualitative (life history interviews)	7	Acquired/traumatic physical disability (Paralympic athletes)	N/R	N/R	Multiple (athletes preparing for London 2012 Paralympic Games)	Entry/Initiation	Posttraumatic Growth theory	Meaning-making following trauma; initial physical activity experiences as a catalyst for posttraumatic growth	Observation of sport participation supplementing interview data	Life history interviews; holistic content analysis	Three main themes reflected participants' initial experiences of becoming physically active after disability, illustrating the role of early physical activity experiences in promoting posttraumatic growth among Paralympic athletes with acquired disabilities	1 Qualitative	3/5
46	Clutterbuck, Auld & Johnston, 2022	Australia	Quantitative (randomized controlled trial)	54	Cerebral palsy (ambulant children; GMFCS I–II)	6–12 years	35.2% (19/54)	Multi-sport peer-group intervention (Sports Stars)	Entry/Initiation	Physical Literacy framework (companion program literature)	Not the primary focus; the intervention targeted sport-specific gross-motor activity, confidence, teamwork, and participation outcomes.	Practitioner-led, peer-group intervention format; eight weeks of physiotherapist-led sport-specific training, sport education, teamwork development, and confidence building	Randomized controlled trial; activity and participation outcome measures	Randomized controlled trial of the eight-week Sports Stars practitioner-led, peer-group sports intervention for 54 ambulant children with cerebral palsy, evaluating activity and participation outcomes	2 Randomized controlled trial	≤2/5
47	Alarfaj, Hassan, Aljohar, Almuaili & Hassan, 2025	Saudi Arabia	Qualitative (case study)	3	Physical disability (twice-exceptional Paralympic champions)	mean 33 years	0%	Multiple (Paralympic sports)	Indirect/Prospective Maintenance-Related Evidence	Self-Determination Theory (intrinsic motivation implied)	Intrinsic motivation and self-determination as key drivers of pursuing/maintaining Paralympic participation		Case study methodology	Investigated developmental factors influencing performance excellence in twice-exceptional (gifted + disabled) Saudi Paralympic champions, emphasizing the pivotal role of intrinsic motivation and self-determination in sustained elite participation	1 Qualitative	≥4/5
48	Yılmaz, Gümüşay & Akkaya, 2021	Türkiye	Quantitative descriptive	57	Physical disability (elite athletes)	N/R	15.8% (9/57)	Multiple (elite parasport)	Current/General Participation	Self-Determination Theory (intrinsic/extrinsic motivation, amotivation)	Intrinsic and extrinsic motivation; amotivation (lack of competence/control sense)	External rewards, appreciation, and societal praise (extrinsic motivation sources)		Examined elite physically disabled athletes' motivation levels for sport participation, situating findings within self-determination theory's intrinsic/extrinsic/amotivation framework	4 Quantitative descriptive	≤2/5
49	Ballas, Buultjens, Murphy & Jackson, 2022	Australia	Qualitative	10	Physical impairment (grassroots-to-elite athletes)	N/R	N/R	Multiple (grassroots to elite para sport)	Entry/Initiation; Observed Behavioral Persistence	Self-Determination Theory; Social Model of Disability	Well-being gains: improved fitness, social interaction, self-confidence	Primary school and high school identified as highly influential entry environments; these settings viewed as platforms for inclusive culture and disability education for the wider community	Interviews; thematic analysis	Examined sporting experiences of 10 high-performing Australian athletes with physical impairment from grassroots to elite level; found well-being increases (fitness, social interaction, self-confidence) and identified school environments (primary/high school) as key influential settings for establishing sport interest	1 Qualitative	≥4/5
50	Orr, Tamminen, Sweet, Tomasone & Arbour-Nicitopoulos, 2018	Canada	Qualitative	8	Physical disability (youth)	Youth	N/R	Team sport (general, peer-focused)	Current/General Participation	Self-Determination Theory; peer relationship framework (friendship/acceptance/victimization, Ladd et al. 1997)	Peer need-supporting vs. need-thwarting behaviors as motivational/psychological factors	Sport peers broadly defined (large age range, all abilities); dynamic peer roles across participation stages	Relational mapping technique; interviews; deductive thematic analysis	Guided by self-determination theory, this study found youth with physical disability perceived their sport peers to hold dynamic roles across three types (friendship, acceptance, victimization/need-thwarting)	1 Qualitative	3/5

No.	Author(s), Year	Country	Study Design	Sample Size (N)	Disability Type	Age Range	% Female	Sport Type/Level	Participation Process / Evidence Category	Theoretical Framework	Motivational Factors	Contextual Factors	Measurement Instruments	Key Findings	MMAT Category	Consensus MMAT Criteria Met (descriptive only)
														throughout their sport participation		
51	Sousa Junior, Clutterbuck, Guimaraes, Souza, Silva, Wright, Camargos & Leite, 2026	Brazil	Quantitative (RCT)	38	Cerebral palsy (ambulant)	6-12 years	N/R	Modified multi-sport intervention (Sports Stars Brazil)	Entry/Initiation; Indirect/Prospective Maintenance-Related Evidence	None stated directly (Physical Literacy framework, per the Sports Stars program lineage—R0183/R0273/R0802)	Participation goals and activity competence as intervention targets	Modified/adapted version of the practitioner-led peer-group Sports Stars program for the Brazilian context	Free-time physical activity measurement; movement-skill assessment; everyday-activity involvement measures; 3-month follow-up	In a randomized controlled trial, children with CP receiving the modified Sports Stars Brazil program showed better improvement in free-time physical activity and movement skills than those receiving usual therapy alone; gains persisted at 3-month follow-up	2 Randomized controlled trial	≤2/5
52	Freitas, Oliveira, Arruda, Oliveira & Oliveira, 2021	Brazil	Quantitative descriptive (comparative/correlational)	27	Physical disability (adapted athletics/para atletismo athletes; acquired vs. congenital disability groups compared)	mean 34.93 (SD 8.51)	N/R	Para athletics (adapted athletics)	Current/General Participation	None stated (EMI-2 exercise-motivation constructs)	Social recognition and competition (differed significantly between acquired- and congenital-disability groups); disease prevention, health/well-being, and fitness (most frequent motives overall, especially in congenital-disability group)	Disability etiology (acquired vs. congenital) and duration/age examined as moderating factors	Exercise Motivations Inventory (EMI-2)	Compared and correlated reasons for adherence to para-athletics among 27 adults with physical disabilities, subdivided by acquired vs. congenital disability; social recognition correlated with disability duration; disease prevention correlated with age; health, well-being, and competition motives were most salient overall, moderated by age and disability etiology	4 Quantitative descriptive	≤2/5
53	Asare, Townsend & Burrows, 2023	New Zealand/UK	Qualitative	12	Physical impairment (athletes, various types)	N/R	N/R	Disability sport (multiple, assistive-technology-dependent)	Current/General Participation	None stated (disability studies/embodiment theory)	Sense of embodied freedom; self-construction and identity via assistive technology (AT) use	Learning-to-use process for AT; dimensions of access required to fully utilize AT in sport	Semi-structured interviews	Centered the experiences of 12 athletes with physical impairments using assistive technology (AT) for sport participation, illustrating how AT shapes embodied freedom, self/other construction, and identifying access dimensions needed to fully utilize AT	1 Qualitative	≥4/5
54	Hernández-Lanas, Bruna-Torres, Carrasco-Palacios, Lucero-Sánchez & Molina-Pacheco, 2024	Chile	Qualitative	4	Physical disability	25–39 years	0%	Adapted sports (general)	Current/General Participation	None stated (occupational participation framing)	Perception of assistive technology as influencing occupational participation	Widespread use of assistive technology in adapted sport as an environmental/equipment factor		Explored perceptions of individuals with physical disabilities regarding assistive technology and its influence on their occupational participation in adapted sports	1 Qualitative	3/5
55	Cheung, Buren, Benn, Alton, Craven, Marzolini & Musselman, 2025	Canada	Mixed methods (case series)	5	Spinal cord injury or disease (aging adults, 50+)	≥50 years	20% (1/5)	Handcycling (remote/telehealth-delivered)	Entry/Initiation	None stated		Peer facilitation; remote/telehealth delivery addressing transportation barriers (a documented top barrier to sport participation)	Concurrent mixed-methods case series design	Examined feasibility and effect of a peer-facilitated, remote handcycling sport program on physical, psychological, and social health of aging adults with SCI/D, explicitly designed to overcome transportation barriers via telehealth delivery	5 Mixed methods	≥4/5
56	Sarol, 2024	Türkiye	Qualitative (phenomenological)	13	Physical disability (wheelchair basketball players)	N/R	N/R	Wheelchair basketball	Current/General Participation	None stated (constraint-negotiation framing implied)	Motivational factors underlying participation; overcoming personal/psychological constraints	Social acceptance and disability status acceptance via sport; peer competition opportunities as facilitators (per literature review framing)	Phenomenological interviews	Phenomenological study determining motivational factors in wheelchair basketball participation and how players overcame constraints to participation, situating findings within the broader literature on psychological, social, and physical benefits of disability sport	1 Qualitative	≥4/5
57	Bates, Kearns, Witten & Carroll, 2019	New Zealand	Qualitative (case study)	6 interviewees (3 players; 3 coordinators)	Physical disability (youth wheelchair basketball team members)	player interviewees 12–22 years	N/R	Wheelchair basketball	Current/General Participation	'Enabling Places' concept (Duff, 2011); disability geography	Identity and independence formation during transition to adulthood; wellbeing	Wheelchair basketball court/team space as an 'enabling place' counteracting social marginalization; analyzed at three spatial scales (body/wheelchair; court; team/club)	Interviews with team members and coordinators; narrative spatial analysis	Investigated how wheelchair basketball creates 'enabling places' that enhance young people's wellbeing, analyzing participants' narratives across three spatial scales (the body and wheelchair, the court, and the wider team context) using a case study of an Auckland, NZ	1 Qualitative	≤2/5

No.	Author(s), Year	Country	Study Design	Sample Size (N)	Disability Type	Age Range	% Female	Sport Type/Level	Participation Process / Evidence Category	Theoretical Framework	Motivational Factors	Contextual Factors	Measurement Instruments	Key Findings	MMAT Category	Consensus MMAT Criteria Met (descriptive only)
														youth wheelchair basketball team		
58	Cheung, Benn, Buren & Musselman, 2026	Canada	Qualitative descriptive	5	Spinal cord injury (aging adults)	aging adults	N/R	Remote/telehealth-delivered sport program (general)	Indirect/Prospective Maintenance-Related Evidence	Integrated Knowledge Translation (IKT) partnership approach	Sense of partnership/collective ownership ('the we') in program design	Continuous/ongoing program delivery as a structural facilitator; peer partnership in program design, implementation and evaluation	Codebook thematic analysis of interview data (5 participants, 3 program leaders)	Three themes: (1) the journey as a collaborative 'we' (research partnership/teamwork), (2) participant preferences/feedback on program structure and equipment, and (3) the need for continuous, ongoing sport programming beyond the research phase; IKT partnership approaches can mitigate barriers and promote ongoing sport participation for aging adults with SCI	1 Qualitative	≥4/5
59	van Oorschot, van Ee & Keijsers, 2023	Netherlands	Quantitative (pre–post intervention, short communication)	13	Lower limb amputation	median 51 years (IQR 40–63)	N/R	Multiple (soccer, sitting volleyball, wheelchair basketball, boxing, golf) via 'Amputee Parateam' program	Entry/Initiation	None stated		Two-part structured intervention (rehabilitation-center fitness phase + multi-sport clinic phase at professional soccer club facility); social support/peer contact via fixed annual group format addressing social barriers (lack of peers, shaming practice among able-bodied individuals)	Physical functioning measures (pre/post); sports physician and prosthetist clearance procedures	The 'Amputee Parateam' program (fitness phase + multi-sport exploration phase across 5 sports) was designed to address physical, environmental, and social barriers to sport participation for lower-limb amputees, improving physical functioning and sports participation	3 Non-randomized	≤2/5
60	Clutterbuck, Gent & Thomson, 2025	Australia	Qualitative	14	Cerebral palsy (ambulant, school-aged children—parent perspective)	school-aged (parent-reported)	N/R	Multiple team sports (Sports Stars: 4 sports introduced over 8 weeks)	Indirect/Prospective Maintenance-Related Evidence	None stated explicitly (Physical Literacy framework underlies the parent Sports Stars program, per R0183/R0273)	Long-term motivation/interest ('spark') following intervention exposure	Staged skill-progression design; practitioner-led peer-group format; introduction of multiple sports sequentially	Parent interviews/qualitative exploration of long-term participation perceptions	Explored parent perceptions of long-term sports participation after children with CP completed the practitioner-led, peer-group Sports Stars intervention, examining what 'sparked' sustained interest beyond the program itself	1 Qualitative	3/5
61	Javorina, Shirazipour, Allan & Latimer-Cheung, 2020	Canada	Qualitative (phenomenological)	33	Acquired physical disability	19–73 years (civilian); 30–68 years (military)	27.3% (9/33)	Adapted physical activity (general)	Entry/Initiation	Psychosocial/disability model integrating structural barriers, psychological well-being, and social relationships	Psychological well-being (self-esteem) as shaped by social relationships and structural barriers	Social relationships (peer support, autonomy support) supporting initiation; accessibility barriers (e.g., recreational building access) leading to exclusion	Phenomenological interviews interpreting lived experience	Examined the impact of social relationships on initiation into adapted physical activity for individuals with acquired disabilities, highlighting how peer/autonomy support and structural accessibility barriers jointly shape psychological well-being and entry into activity	1 Qualitative	3/5
62	Kong, Quek, Liu, Ma & Tan, 2026	Singapore	Mixed methods (case study)	4	Physical disability (novice athletes, standing and wheelchair users)	N/R	N/R	Table tennis	Entry/Initiation	Constraints-Led Approach (CLA, motor learning/coaching pedagogy)	Not the primary focus (study centers on coaching design)	Customized/tailored coaching strategies as a facilitator; equipment/rules structure allowing standing and wheelchair players to compete together; CLA's flexibility for diverse disability needs	Eight one-to-one training sessions per participant; case-study observation/performance tracking	Designed and implemented customized, Constraints-Led-Approach-informed table tennis coaching for 4 novice players (2 standing, 2 wheelchair users) with physical disabilities across 8 one-to-one sessions, addressing a gap in coaching-pedagogy research for this population (as opposed to intellectual disability, more commonly studied in para-table tennis)	5 Mixed methods	≤2/5
63	Amberkar, Agarwal, Singh, Shete & Mullerpatan, 2019	India	Quantitative descriptive (survey, embedded in a narrative review)	102	Spinal cord injury	mean 40.41	14%	Sport (general, paraplegic sports)	Withdrawal/Dropout/Nonparticipation	None stated	Self-motivation (lack thereof cited as a top reason for non-participation, alongside poor fitness and low confidence)	Family support; financial security; institutional support (motivation and training facilities) as top facilitators; difficulty reaching sports grounds and lack of disabled-friendly toilets/accommodation as top barriers	Interview-based survey (4 paraplegic rehabilitation centers, Mumbai)	Among 102 adults with SCI in Mumbai, India, 60% (61/102) participated in sports; family support, financial security, and institutional support were top facilitators; physical accessibility (reaching grounds, toilets, accommodation) was top barrier; lack of self-motivation, poor fitness, and low confidence were the main reasons for non-participation	4 Quantitative descriptive	3/5

No.	Author(s), Year	Country	Study Design	Sample Size (N)	Disability Type	Age Range	% Female	Sport Type/Level	Participation Process / Evidence Category	Theoretical Framework	Motivational Factors	Contextual Factors	Measurement Instruments	Key Findings	MMAT Category	Consensus MMAT Criteria Met (descriptive only)
64	Ćorić & Ljubotina, 2013	Croatia	Quantitative descriptive	101	Physical disability (war veterans)	N/R	0%	Sitting volleyball	Entry/Initiation	None stated (psychosocial reintegration framing)	Friendship and health concern as main participation motives; emotional benefits (physical/mental health, social life, general usefulness) rated highly by 75-85% of participants	Recruitment via friend invitation (common entry pathway); family/friends' understanding and appreciation rated higher than institutional/state support	Quality of Life Questionnaire; motives/participation reasons questionnaire (instrument not fully named in visible excerpt)	Among 101 Croatian war veterans with physical disability playing sitting volleyball, most began via friend invitation; friendship and health concern were the main motives; 75-85% reported high perceived benefit to physical/mental health and social life; overall quality of life was comparable to the general male population, with better material/leisure satisfaction but worse mental health; family/friends were more supportive than institutions/state	4 Quantitative descriptive	≤2/5
65	Prokopowicz, Molik, Prokopowicz, Chamera, Ogonowska-Słodownik, Lencse-Mucha, Morgulec-Adamowicz, Kosmol & Perkowski, 2016	Poland	Quantitative descriptive (comparative)	52	Physical disability (motor/lower-limb, Paralympic sailors)	N/R	N/R	Paralympic sailing (regatta: Sonar, 2.4mR, Skud 18 classes)	Current/General Participation	None stated (Participation Reason Scale, Brasile & Hedrick 1991)	Emotions related to physical activity and opportunity for skill development (top motives for Polish national team sailors); being with friends and the challenge of sailing (top motives for other European sailors)	National/team context (Polish National Team vs. sailors from 10 other European countries) as a comparative cultural factor	Participation Reason Scale (PRS; Brasile & Hedrick, 1991)	Compared participation motives between 20 Polish National Team sailors with disabilities and 32 sailors from 10 other European countries at Paralympic sailing events; Polish sailors rated activity-related emotions and skill development highest, while other European sailors rated friendship and challenge highest	4 Quantitative descriptive	≤2/5
66	Swanson, Colwell & Zhao, 2008	USA	Quantitative descriptive (comparative)	193	Physical disability (wheelchair athletes)	13-34 years	31%	Wheelchair sports (general)	Current/General Participation	None stated (motivation and social-support taxonomies)	Four motivation sources: escape, self-esteem enhancement, self-improvement, social interaction	Six forms of social support: emotional challenge, emotional support, listening support, reality confirmation, task appreciation, task challenge	Motivation and social support inventories	Explored relationships among four motivation sources and six social-support forms among 193 wheelchair athletes (133 male, 60 female, 13-34y), examining differences by gender, age, playing level, skill level, years of participation, and future playing intentions; males were more motivated than females by escape and	4 Quantitative descriptive	3/5
67	Field & Oates, 2001	Australia	Quantitative descriptive (survey, comparative)	166	Spina bifida (n=97) and cystic fibrosis (n=69)—spina bifida subgroup separately reported	children	N/R	Not sport-specific (general sport/recreation opportunities)	Current/General Participation	None stated	Family stress/balancing needs of disabled and non-disabled siblings (psychosocial context)	Lack of variety in available activities; difficulty finding family-inclusive activities; disability-specific barriers to participation	Parent-report survey	Opportunities for sport/recreation participation were significantly greater for children with cystic fibrosis than for children with spina bifida. Parents of both groups reported insufficient variety of activities, difficulty finding family-inclusive options, and barriers to their child's participation	4 Quantitative descriptive	≤2/5
68	Namai, Jung, Shaw & Martin Ginis, 2025	Japan (research team based in Canada/Japan)	Qualitative (abductive; COM-B framework)	9	Spinal cord injury	mean 52 years (SD 13)	44.4% (4/9)	Exercise and sport (general)	Current/General Participation	COM-B model (Capability–Opportunity–Motivation–Behavior)	Individual motivation examined within COM-B framework, contextualized by Japan's distinct fitness/exercise culture	Cultural stigma/shame around disability in Japan (managed within family, medical-model framing); limited fitness/exercise culture compared to Western countries; accessibility/social inclusion shaped by these attitudes	Interviews; abductive analysis (inductive then deductive mapping to COM-B model)	Explored factors influencing exercise and sport participation among Japanese adults with SCI, situating findings within Japan's distinct disability-stigma and fitness-culture context using the COM-B behavioral model and abductive analysis	1 Qualitative	≥4/5
69	Ali & Rider, 2024	USA	Qualitative (grounded theory)	12	Spinal cord injury	N/R	N/R	Quad rugby	Entry/Initiation; Indirect/Prospective Maintenance-Related Evidence	Grounded theory (no substantive theory named)	Motivation for continued participation; benefits of participation	Enabling factors for quad rugby participation; barriers to participation	Semistructured interviews	Four themes: benefits of quad rugby participation, enabling factors, barriers, and motivation for continued participation; highlights importance of early introduction to quad rugby after SCI and biopsychosocial benefits; occupational therapists can address barriers via innovative approaches/advocacy	1 Qualitative	≥4/5

No.	Author(s), Year	Country	Study Design	Sample Size (N)	Disability Type	Age Range	% Female	Sport Type/Level	Participation Process / Evidence Category	Theoretical Framework	Motivational Factors	Contextual Factors	Measurement Instruments	Key Findings	MMAT Category	Consensus MMAT Criteria Met (descriptive only)
70	Moll & Bester, 2019	South Africa	Quantitative descriptive	140	Mobility impairment (adolescents, boys and girls)	adolescents	N/R	Not sport-specific (general sport participation vs. nonparticipation)	Withdrawal/Dropout/Nonparticipation	None stated	Emotions; relationships with parents and peers	Age and gender; school type; hostel dwelling (structural factors)	Author-developed factor survey (regression/discriminant analysis, 22% variance explained)	Four factors (spanning personal, structural, and relational domains) explained 22% of the variance distinguishing sport participants from non-participants among adolescents with mobility impairments	4 Quantitative descriptive	≤2/5
71	Sit, Lindner & Sherrill, 2002	Hong Kong (China)	Quantitative descriptive	237	Physical disability (school-aged children, 5 disability types)	9-19 years	N/R	Sport (excluding physical education classes)	Current/General Participation	None stated		Gender (girls significantly less active than boys); school level; disability type; cultural context (special-school system in Hong Kong vs. Western mainstream settings)	Sport participation questionnaire (individual interviews)	Among 237 Hong Kong Chinese children with disabilities (9-19y) in special schools, girls were significantly less active than boys; participation frequency and extent varied by school level and disability type, extending Western findings to an Asian cultural context	4 Quantitative descriptive	≥4/5
72	Tasiemski, Bergström, Savic & Gardner, 2000	United Kingdom (Poland-based lead author)	Quantitative descriptive (pilot postal survey)	45	Spinal cord injury (C5 or below, wheelchair-dependent)	18-50 at injury	N/R	Sports and recreation (general)	Current/General Participation	None stated	Not the primary focus (study examines associations with education/employment)	Association between sport/recreation involvement and education/employment levels	Postal survey (sport/recreation, education, employment questions)	Pilot study examining whether involvement in sport and recreation among people with SCI is associated with higher levels of education and employment, based on a postal survey of 45 individuals at a UK spinal injuries center	4 Quantitative descriptive	≤2/5
73	Qi, Cai & Meng, 2021	China	Qualitative	5	Physical disability (children)	children	40%	Multiple (Paralympic-Games-themed workshops)	Current/General Participation	None stated (beliefs/attitudes framing)	Beliefs about sport/PA engagement; motivational impact of Paralympic Games exposure	Workshop-based exposure to the Paralympic Games as a structured intervention/context	Individual semi-structured interviews (pre/post workshop)	Explored beliefs of Chinese children with physical disabilities regarding sport/PA engagement, and how exposure to Paralympic Games content (via workshops) impacted these beliefs, addressing a gap in non-Western Paralympic-legacy research	1 Qualitative	≥4/5
74	Coates & Vickerman, 2016	United Kingdom	Qualitative	8	Physical disability (adolescents)	11-16 years	N/R	Multiple (Paralympic-Games-related motivation, not sport-specific)	Current/General Participation	None stated (legacy/role-model framing)	Paralympians as role models; motivating nature of the Paralympic Games (inspiration); changing self-perceptions	Paralympic Games media/cultural exposure as a societal-level contextual factor (changing perceptions of disability)	Semi-structured interviews; thematic analysis	Three themes described the Paralympic legacy for children with disabilities: Paralympians as role models, changing perceptions of disability, and the motivating nature of the Paralympics; the London 2012 Games were inspirational and improved participants' self-perceptions	1 Qualitative	3/5
75	Ojwang, Foster & Odiango, 2025	Kenya	Qualitative	5	Physical disability (elite para-athletes)	N/R	60% (3/5)	Multiple (Kenyan elite para-sport)	Current/General Participation	Constraints framework (Crawford & Stodolska, 2008): basic, intermediate, fundamental constraints	Personal-level negotiation of basic constraints as a resilience/agency factor	Basic constraints (individually negotiable); intermediate constraints (societal, addressable collectively); fundamental constraints (deeply embedded societal barriers, hardest to overcome) — a tiered structural-barrier framework	Qualitative interviews (lived-experience methodology)	Explored lived experiences of Kenyan para-athletes using a tiered constraints framework (basic/intermediate/fundamental), reframing barriers as possibilities and addressing why developing countries show low Paralympic participation/success	1 Qualitative	3/5
76	Hayward, Hogan, Melam, Raine, McCullough & Bell, 2025	USA/Scandinavia (multi-national sample)	Mixed methods (qualitative case study + physiological/QoL measures)	10	Physical disability (power wheelchair users)	adults	N/R	Power wheelchair soccer and volt hockey	Current/General Participation	None stated	Player experience and engagement with adaptive team sport	Availability of adaptive/inclusive programs for power wheelchair users (noted as scarce); social support	Qualitative interviews (10, across 5 US + 5 Scandinavian players); WHOQOL-BREF; heart rate monitoring during play	Multi-method case study of power wheelchair adaptive team sport (soccer, volt hockey) describing player experience, quality of life (WHOQOL-BREF), and physical fitness (heart rate during play)	5 Mixed methods	≤2/5
77	Kim, Thomas, Sharpe & Farrell, 2025	USA	Qualitative	104	Physical disability	male mean 41.56; female mean 36.90	27.9% (29/104)	Adaptive sports (general, campus recreation context)	Current/General Participation	None stated		Limited availability of adaptive/inclusive campus recreation programming; lack of staff knowledge of accommodations leading to participant hesitation to explore opportunities		Explored reasons for participation and barriers within adaptive sports for individuals with physical disabilities, centering athlete/participant perspectives—a gap identified in prior benefit-focused (rather than perspective-focused) research, particularly in the campus-recreation context	1 Qualitative	≥4/5
78	Kim, Kang & Seo, 2005	Korea (international sample; study	Quantitative descriptive	79	Spinal cord injury	15.9–73.1 (mean 40.2)	20%	Para shooting	Entry/Initiation	None stated	Self-motivation (39.3%); disabled friends (21.5%);	Medical staff encouragement (20.2%);	Researcher-developed structured interview;	Self-motivation and peer (disabled-friend) influence	4 Quantitative descriptive	3/5

No.	Author(s), Year	Country	Study Design	Sample Size (N)	Disability Type	Age Range	% Female	Sport Type/Level	Participation Process / Evidence Category	Theoretical Framework	Motivational Factors	Contextual Factors	Measurement Instruments	Key Findings	MMAT Category	Consensus MMAT Criteria Met (descriptive only)
		conducted in South Korea)						(elite/international)			coach/other athletes (12.7%); family (6.3%)	facility/venue accessibility (9.9% barrier); financial difficulty (6.3% barrier); physical barriers (spasticity, neurogenic pain, bladder control, muscle weakness)	international functional classification exam	were the leading motive providers for starting shooting; main perceived benefits were self-confidence, friendship, independence, and social participation; main barriers were physical symptoms during competition, facility accessibility, and cost		
79	Feiten, Schaab & Sanfelice, 2025	Brazil	Qualitative	14	Physical disability (multiple types, Paralympic athletes)	N/R (varied ages)	N/R (both sexes)	Multiple individual Paralympic sports	Current/General Participation	None stated (Bardin content analysis used methodologically)	Personal acceptance/identity transformation; empowerment; sense of belonging and citizenship	New social interactions; financial, cultural and educational opportunities; accessibility barriers; regional resource inequality; precarious professionalization; media representation	Semi-structured narrative interviews (individual); Bardin categorical content analysis	Paralympic sport positively contributed to personal acceptance and favored new social interactions, plus financial/cultural/educational opportunities; persistent structural challenges: accessibility barriers, regional resource inequality, precarious professionalization, and media representation; sport reframed disability, strengthening autonomy and public recognition	1 Qualitative	3/5
80	Ortiz Cárdenas, Perdomo Vargas, Garzón Pastrana & Vargas Reyes, 2021	Colombia	Quantitative descriptive	49	Physical disability (female Para Powerlifting athletes)	N/R	100%	Para Powerlifting (elite/international)	Current/General Participation	None stated	Perceived opportunity for professional sport development	Lack of adequate sport venues/facilities; insufficient disability- and sport-specific adaptation; government-level measures taken but insufficient for female para-sport growth	Survey (perception of barriers/facilitators)	Para Powerlifting was perceived as an important motivator due to professional development opportunities; key barriers: lack of appropriate sport venues and specific adaptation to disability/sport needs; despite some government action, further work is needed to grow female para-sport participation	4 Quantitative descriptive	≤2/5
81	Stephens, Neil & Smith, 2012	United Kingdom	Qualitative	7	Spinal cord injury	N/R	N/R	Multiple (variety of sports)	Current/General Participation	None stated (thematic analysis)	Self-worth; physical challenge; emotional benefits (e.g., emotional outlet)	Organizational barriers (facility availability); medical barriers (e.g., UTIs); lack of information; views held by others; socialization as a benefit	Semi-structured interviews	20 benefits and 18 barriers identified; benefits grouped into socialization, self-worth, physical challenge, and emotional sub-themes; barriers grouped into organizational, medical, emotional, lack-of-information, and others' views sub-themes	1 Qualitative	3/5
82	Sá, Azevedo, Martins, Machado & Tavares, 2012	Portugal	Quantitative descriptive (survey + facility audit)	24 participants; 11 public sports facilities	Reduced mobility (general)	25–42 years (mean 33)	N/R	Not sport-specific (general sport facility access)	Current/General Participation	None stated	Motivation and perceived benefits of sport (assessed via questionnaire section 3)	Accessibility of sports facilities (checklist based on Portuguese/EU legislation); recruitment via Rehabilitation Centers and Physiotherapy Clinics in greater Porto area	Facility accessibility checklist (Portuguese norms/EU directives); 3-part questionnaire (personal data; physical activity; motivation and benefits)	Combined facility-accessibility audit with a participant survey on motivation/benefits, aiming to explain persistently low sport-participation rates among people with reduced mobility in the Porto area and raise awareness of accessibility needs among providers	4 Quantitative descriptive	≤2/5
83	Comaduran Marquez, Vaandering, Babwani, Redquest, Nikitovic, Kelly, Kinney-Lang & Kirton, 2026	Canada	Mixed methods	6	Quadriplegic cerebral palsy (children)	children	N/R	Boccia (BCI-leveraged/adapted technology)	Current/General Participation	None stated	Keen interest in using BCI technology to access sport (child-reported)	Current participation challenges; systemic/technological facilitators (BCI system access); caregiver-reported barriers and facilitators	Semi-structured caregiver interviews (n=6); new 21-item child feedback survey (n=6)	Identified current participation challenges and facilitators to sport for children with quadriplegic CP, and explored future possibilities/benefits of brain–computer-interface (BCI)-leveraged Boccia; children expressed strong interest in using BCI to access sport	5 Mixed methods	≤2/5
84	Barbosa de Lima & de Jesus Alves, 2020	Brazil	Qualitative (case study)	3	Physical disability (adolescents)	12-18 years	N/R	Bocce and Para-badminton	Current/General Participation	Matching Person and Technology (MPT) model	Not the primary focus (study centers on assistive technology matching)	Assistive technology (AT) choice, prescription, and follow-up process as an environmental/occupational-therapy-mediated facilitator	Characterization Questionnaire; Quebec User Evaluation of Satisfaction with Assistive Technology (B-QUEST); Assistive Technology assessment (MPT-based)	Analyzed the process of choosing, prescribing, and following up on assistive technology for 3 adolescents in competitive Bocce and Para-badminton using the Matching Person and Technology model, aiming to reduce environmental barriers to sport participation	1 Qualitative	≤2/5
85	Berardi, Galeoto, Lucibello, Panuccio,	Italy	Quantitative descriptive (cross-sectional)	128	Physical disability (athletes using sport wheelchairs)	mean 30.55 years (SD 11.5)	14.1% (18/128)	Multiple (sport wheelchair users)	Current/General	None stated	Not the primary focus (equipment satisfaction is the focus)	Sport wheelchair satisfaction/equipment quality as an	Satisfaction questionnaire (instrument not fully named in visible excerpt, likely QUEST-	Cross-sectional Italian study assessing athletes with disabilities' satisfaction with their sport wheelchairs, an	4 Quantitative descriptive	3/5

No.	Author(s), Year	Country	Study Design	Sample Size (N)	Disability Type	Age Range	% Female	Sport Type/Level	Participation Process / Evidence Category	Theoretical Framework	Motivational Factors	Contextual Factors	Measurement Instruments	Key Findings	MMAT Category	Consensus MMAT Criteria Met (descriptive only)
	Valente & Tofani, 2021								Participation			environmental/equipment-related facilitator or barrier	based given AT literature convention)	equipment-related factor relevant to sustained sport participation		
86	Hernández-Lanas, Molina-Pacheco, Lucero-Sánchez, Carrasco-Palacios & Bruna-Torres, 2026	Chile	Quantitative descriptive (cross-sectional)	17	Motor disability (boccia athletes)	18+ (35-44 most common)	N/R	Boccia (club-based, ≥6 months practice)	Entry/Initiation; Indirect/Prospective Maintenance-Related Evidence	None stated (occupational participation framing)	Recommendation from others as the primary motivation for starting boccia	Club training environment (reinforces commitment); economic barriers and limited infrastructure (affect sustainability of participation)	Structured questionnaire; SPSS analysis	Characterized occupational participation of 17 athletes with motor disabilities practicing boccia across 3 clubs in the Santiago Metropolitan Region, Chile; most athletes were male, aged 35-44, and began boccia following a recommendation; club training reinforced commitment, but economic barriers and limited infrastructure affected sustainability	4 Quantitative descriptive	≤2/5
87	Poonsiri, Dekker, Dijkstra, Hijmans & Geertzen, 2021	Netherlands	Quantitative descriptive (predictive)	207	Lower limb amputation	mean 62.0 (SD 13.0)	29%	Cycling	Current/General Participation	None stated		Prosthesis type/satisfaction; amputation level (most frequent: transtibial); individual characteristics as cycling predictors	Questionnaire (prosthesis, individual characteristics, amputation, cycling barriers/facilitators, prosthetic satisfaction); logistic regression for predictors	Evaluated cycling participation and identified barriers/facilitators among 207 Dutch adults with lower limb amputation (71% male, mean age 62); variables significantly associated with cycling (p<0.1) were entered into logistic regression to identify predictors	4 Quantitative descriptive	≥4/5
88	Bragaru, Meulenbelt, Dijkstra, Geertzen & Dekker, 2013	Netherlands	Quantitative descriptive (cross-sectional survey)	780	Lower limb amputation	mean 59.6 years (SD 14.8)	38%	Sport participation (general; athlete vs. non-athlete comparison)	Withdrawal/Dropout/Nonparticipation	None stated	Sport-participation status; personal and health-related characteristics associated with participation	Older age, smoking, and a vascular cause of amputation were negatively associated with sport participation; demographic and amputation-related factors were examined.	Postal survey of Dutch lower-limb amputees; multivariable statistical analysis	Of 780 analyzable respondents, 15% participated in sport for at least 5 hours per month; older age, smoking, and a vascular cause of amputation were negatively associated with sport participation	4 Quantitative descriptive	≥4/5
89	Nettleton, Hassett, Scheibe, Price, Kirkham & Sherrington, 2017	Australia	Quantitative—cohort (longitudinal)	39	Physical disability (adults attending disability sport exhibition)	adults	N/R	Multiple (disability sport exhibition attendees)	Observed Behavioral Persistence	None stated	Desire for greater sport involvement despite low overall activity	Cost (most common barrier at baseline, 54%); lack of appropriate sporting competitions (most common at 6-month follow-up, 50%)	Physical Activity Scale for Individuals with a Physical Disability (PASIPD); modified Barriers to Physical Activity and Disability Survey (B_PADS)	At baseline, 64% played sport but were physically inactive overall; at 6-month follow-up (n=32, 82% retention), 66% played sport with still-low physical activity levels; cost was the top barrier at baseline; lack of appropriate competitions was the top barrier at follow-up; authors call for increased opportunities and barrier reduction	3 Non-randomized	≤2/5
90	Ayyildiz, Aras, Yagin, Gülü, Yapici & Alwhaibi, 2024	Türkiye	Quantitative descriptive (cross-sectional)	253	Physical disability and hearing impairment (physically disabled subgroup relevant)	18-47	N/R	Multiple (not sport-specific)	Current/General Participation	None stated	Health and social interaction identified as most important motivators; feeling of freedom through sport (from cited Turkish literature)	Time constraints, cost, and lack of opportunity identified as key barriers; insufficient government policy/trainer support for people with disabilities	Sports participation motivation questionnaire (instrument not fully named in visible excerpt)	Examined sports participation motivation among 253 physically and hearing-impaired individuals (18-47 years) in Turkey; health and social interaction were the most important motivators, while time, cost, and opportunity were key barriers	4 Quantitative descriptive	≤2/5
91	Dutia, Curran, Donohoe, Beckman & Tweedy, 2022	Australia	Quantitative (comparative, time-motion analysis)	23	Cerebral palsy with high support needs (CP&HSN)	mean ~20 (19.7-21.0)	48%	Para swimming	Current/General Participation	None stated	Not the primary focus (study centers on structural/logistical burden)	Time cost of tasks required for participation (e.g., preparing to commence/depart training)—a novel structural/environmental barrier construct explaining underrepresentation of high-support-needs athletes	Time-motion analysis of training-related tasks; comparison groups (non-disabled n=20, para swimmers with CP&HSN n=3)	Used time-motion analysis to quantify the time cost of tasks required for sports participation among athletes with CP and high support needs (CP&HSN), a population profoundly inactive and underrepresented in physical activity research, comparing them to non-disabled swimmers	3 Non-randomized	≤2/5
92	Scheuer, Anneken, Hirschfeld, Richarz & Thietje, 2009	Germany	Quantitative descriptive (retrospective survey)	309	Spinal cord injury (paralysis)	≤65 years (subgroup analyzed)	N/R	Sport (general, not sport-specific)	Current/General Participation	None stated		Etiology of paralysis (8.9% traumatic, 21.1% disease/other); time since injury onset (~half >5 years); employment status examined as a contextual/socioeconomic factor	Retrospective anonymized questionnaire; chi-square test, t-test, ANOVA	Retrospective anonymized survey study on sporting activity and social/occupational participation among German people with spinal cord injury/paralysis, examining associations with etiology, time since injury, and employment	4 Quantitative descriptive	3/5

No.	Author(s), Year	Country	Study Design	Sample Size (N)	Disability Type	Age Range	% Female	Sport Type/Level	Participation Process / Evidence Category	Theoretical Framework	Motivational Factors	Contextual Factors	Measurement Instruments	Key Findings	MMAT Category	Consensus MMAT Criteria Met (descriptive only)
														status (subgroup of 309 respondents ≤65 years old)		
93	Faraji, Dabiri, Allami, Hosseini & Barati, 2023	Iran	Quantitative (secondary data analysis)	2644	Extremity musculoskeletal disorders (war veterans)	N/R	N/R	Not sport-specific (general sport/physical activity participation)	Withdrawal/Dropout/Nonparticipation	None stated		Type of extremity disorder (highest participation, 65%, among bilateral upper limb amputees) as a structural/physical factor	Secondary analysis of health needs assessment data (2005-2017); chi-square, independent t-test, partial correlation, binary logistic regression	Among 2,644 Iranian veterans with extremity musculoskeletal disorders assessed over a 12-year period, 37.6% (994) participated in at least one sport/physical activity; bilateral upper limb amputees showed the highest participation rate (65%)	3 Non-randomized	≤2/5
94	Çelenk, 2021	Türkiye	Quantitative descriptive (comparative)	732	Physical disability (orthopedic subgroup relevant; also includes visual and hearing impairment subgroups reported separately)	N/R	N/R	Not sport-specific (sport-practicing vs. non-practicing compared)	Withdrawal/Dropout/Nonparticipation	None stated	Motivation for physical activity participation (PAPMS) positively correlated with sports' effect on life skills (SLSS)	Disability type (orthopedic, visual, hearing) examined as a moderating/comparative factor	Physical Activity Participation Motivation Scale (PAPMS); Sports' Effect on Life Skills Scale (SLSS)	Among 732 people with physical disabilities (327 sport-practicing, 405 non-practicing), sport-practicing individuals scored significantly higher on both PAPMS and SLSS ($p<0.05$) than non-practicing individuals, with a positive correlation between the two scales; significant differences by disability type were also found for orthopedic, visual, and hearing-impaired subgroups	4 Quantitative descriptive	≤2/5
95	Tekkurşun-Demir & İlhan, 2020	Türkiye	Quantitative descriptive	303	Physical, hearing, and visual disability (federation-licensed athletes; physical-disability subgroup relevant)	N/R	N/R	Multiple (licensed federation sports)	Current/General Participation	None stated	Confidence and psychological stability associated with sport participation (per literature review framing)	Federation-level licensing/institutional structure (Turkey Physical-Hearing-Visual Impaired Sports Federations) as an organizational context	Quantitative survey (sports participation motivation instrument, likely author's own MSSPPD/SPMSDI-family scale given research group)	Determined sports participation motivation among 303 licensed disabled athletes across Turkey's Physical, Hearing, and Visually Impaired Sports Federations, framed within the broader argument that motivation is a foundational element of sport participation and psychological well-being	4 Quantitative descriptive	≤2/5
96	Pérez-Tejero, Borges, Chávez, Pérez-Rodríguez & Coterón, 2023	Spain	Quantitative descriptive (survey)	117	Physical disability (community residents)	15–74 years	33%	Physical sport practice (general, not sport-specific)	Entry/Initiation	None stated		Community/regional context (Madrid)—regional policy/promotion implications	Survey (reasons for sport practice)	Analyzed reasons for physical sport practice among residents of the community of Madrid with physical disabilities, aiming to inform suggestions for promoting active living given PWD's markedly lower PA/sport rates than non-disabled peers	4 Quantitative descriptive	≤2/5
97	Guven, Kara & Ozdedeoglu, 2019	Türkiye	Qualitative	8	Physical disability (women athletes)	N/R	100%	Multiple (not sport-specific)	Entry/Initiation	Socialization theory (sport as socialization mediator)	Socialization into sports (mediating factors prompting entry) and socialization via sports (life effects)	Gendered barriers—the 'double barrier' of disability and gender in the socialization process	Semi-structured interviews (implied)	Explored mediators prompting disabled women's socialization into sport and the effects of sport on their broader socialization ('double barrier' framing), based on interviews with 8 disabled women athletes	1 Qualitative	3/5
98	Alhumaid, Adnan & Said, 2024	Saudi Arabia	Qualitative	20	Physical impairment (women)	19–>40 years	100%	Multiple (Para sports)	Current/General Participation	None stated (empowerment/social inclusion framing)	Empowerment through Para sport participation	Access barriers specific to women with impairments; need for inclusive ecosystem involving government, sports organizations, communities, and individuals collaboratively	Qualitative interviews	Explored empowerment and social inclusion through Para sports among Saudi women with physical impairments, finding that despite access challenges, achieving empowerment requires a collaborative, inclusive ecosystem spanning government, sports organizations, communities, and individuals	1 Qualitative	3/5
99	Anderson, Wozencroft & Bedini, 2008	USA	Quantitative descriptive (comparative)	22	Physical disability (adolescent girls)	10–18 years	100%	Disability sport (multiple)	Current/General Participation	Social support framework	Not the primary focus (social support mechanisms are the focus)	Comparison of social support mechanisms (types/sources of support) supporting adolescent girls' involvement in disability sport	Social support measures (instrument not fully named in visible excerpt)	Compared social support mechanisms underlying adolescent girls' involvement in disability sport, addressing a population (young female athletes with disabilities) that is underrepresented relative to male-focused disability sport research	4 Quantitative descriptive	3/5
100	Darcy, Lewis, Fausett, Neuville, Bennett &	USA/UK (international sample)	Quantitative descriptive (survey)	171	Physical disability (golfers)	mean 51.4 years (SD 12.9)	11.1%	Golf (adaptive)	Current/General Participation	None stated		COVID-19 pandemic-related disruption to physical activity/sport access, compounding pre-	Survey (international sample of golfers with disabilities)	Examined the disproportionate effect of COVID-19-pandemic-related physical activity changes on an international	4 Quantitative descriptive	3/5

No.	Author(s), Year	Country	Study Design	Sample Size (N)	Disability Type	Age Range	% Female	Sport Type/Level	Participation Process / Evidence Category	Theoretical Framework	Motivational Factors	Contextual Factors	Measurement Instruments	Key Findings	MMAT Category	Consensus MMAT Criteria Met (descriptive only)
	Jayabalan, 2024											existing barriers (e.g., obesity and social isolation/loneliness risk factors motivating participation)		sample of golfers with disabilities, in the context of adaptive sports mitigating obesity and social isolation risk		
101	Akhoroz, Erdogan & Yildirim, 2025	USA/Türkiye	Qualitative	11	Physical disability	mean 54.4 years	54.5% (6/11)	Adaptive hockey (volt hockey) and adaptive soccer (power soccer), via bespoke VR training	Current/General Participation	None stated	Perceived potential/benefit of VR-based training tools for engagement and motivation	Design features and technological improvements needed for VR adaptive-sports training (identified via RQ3)	Qualitative exploratory study (interviews/observation, guided by 3 RQs)	Explored how athletes with physical disabilities perceive the potential of bespoke VR games (for volt hockey and power soccer) as adaptive sports training tools, identifying benefits, challenges, and desired design improvements; framed against the context that 51-54% of people with physical disabilities do not participate in leisure-time PA	1 Qualitative	≥4/5
102	Hudson, Sanzo, Zerpa & Klarner, 2024	Canada	Quantitative descriptive (correlational)	21	Transtibial amputation	N/R	29%	Not sport-specific (general sport/PA participation and avoidance)	Current/General Participation	None stated	Balance confidence positively associated with sport participation	Psychosocial adaptation to amputation (implied factor in participation avoidance)	Online survey; Spearman and Pearson correlations	Among 21 people with transtibial amputation, sport participation was positively associated with balance confidence and well-being, with a moderate effect size; addresses a gap in prior lower-limb-amputation research that did not isolate the transtibial subgroup	4 Quantitative descriptive	≥4/5
103	Samsonienė, Adomaitienė, Krivičiūtė, Jankauskienė, Jurkštienė & Kėvelaitis, 2008	Lithuania	Quantitative descriptive (comparative)	35	Severe physical disability (disability group I)	N/R	N/R	Applied physical activity / adapted sport (general)	Withdrawal/Dropout/Nonparticipation	None stated	Self-rated health status (better among sport participants)	Need for assistance from others (lower among sport participants)	Modified Kenny test; researcher-developed questionnaire	Non-sporting disabled individuals rated their health status significantly worse (p=0.02) and reported significantly greater need for assistance from others (p=0.035) than those participating in applied physical activity	4 Quantitative descriptive	≤2/5
104	Schenker, Wolf, Alimusaj, Putz, Lehner & Block, 2025	Germany	Quantitative (prospective comparative)	120	Lower limb amputation (malignant musculoskeletal tumor vs. trauma etiology)	N/R	N/R	Not sport-specific (general sport access and high-level activity)	Current/General Participation	None stated	Not the primary focus (study centers on physical/functional determinants)	Etiology of amputation (tumor vs. trauma) as a structural/medical factor affecting access to high-level activity and sport; comparison against healthy controls and traumatic-amputation group	Timed-Up-and-Go (TUG); 2-Minute-Walk-Test; 10-Meter-Walk-Test; Comprehensive High-Level Activity measure	Compared high-level activity and access to sport between lower-limb amputees due to malignant musculoskeletal tumors versus trauma, hypothesizing tumor patients would show reduced activity/access due to delayed rehabilitation access and cancer-related burden	3 Non-randomized	≤2/5
105	Furst, Ferr & Megginson, 1993	USA	Quantitative descriptive	25	Physical disability (triathlon competitors)	N/R	N/R	Triathlon (National Triathlon for the Physically Challenged)	Entry/Initiation; Observed Behavioral Persistence	None stated	Self-motivation; encouragement from other physically disabled individuals (consistent factor in initial rehabilitation and continued sport participation)	Prior activity before disability onset	Researcher-developed questionnaire	Most competitors had been active before disability onset; encouragement/motivation from other disabled individuals was the consistent factor in both initial rehabilitation-related sport entry and subsequent continued participation	4 Quantitative descriptive	3/5
106	Wu & Williams, 2001	United Kingdom	Quantitative descriptive	143	Spinal cord injury	N/R	N/R	Wheelchair basketball, rugby, tennis, athletics	Entry/Initiation	None stated	Pre-injury sport experience associated with post-injury participation		Disability Sport Participation Questionnaire (author-developed)	Examined relationships between pre- and post-injury sport participation among UK SCI individuals (112 active pre-lesion, 31 inactive pre-injury); identified factors influencing entry into sport after SCI	4 Quantitative descriptive	≥4/5
107	Kirkby, Cull & Foreman, 1996	Australia	Quantitative descriptive	116	Spinal cord injury	N/R	N/R	Wheelchair sports (general)	Entry/Initiation	None stated		Pre-lesion (pre-injury) sports participation history examined as a predictor of post-injury wheelchair sport involvement	Researcher-developed survey; Chi-square analysis	89/116 had prelesion sport involvement, but only 38 (43%) continued after injury; of 27 without prelesion sport history, 13 (48%) became involved post-injury; Chi-square analysis showed pre-lesion sport history was NOT	4 Quantitative descriptive	≤2/5

No.	Author(s), Year	Country	Study Design	Sample Size (N)	Disability Type	Age Range	% Female	Sport Type/Level	Participation Process / Evidence Category	Theoretical Framework	Motivational Factors	Contextual Factors	Measurement Instruments	Key Findings	MMAT Category	Consensus MMAT Criteria Met (descriptive only)
														significantly related to post-injury wheelchair sport involvement		
108	O'Neill & Maguire, 2004	United Kingdom	Quantitative descriptive (survey)	39	Spinal cord injury (incl. Guillain–Barré syndrome)	16-65+ (grouped)	N/R	Multiple (incl. wheelchair rugby, shooting via Interspinal Games)	Entry/Initiation; Observed Behavioral Persistence	None stated	Aspiration to sport (23 patients expressed aspiration despite not currently participating)	Access to sport unavailable locally via Interspinal Games (e.g., wheelchair rugby, shooting); therapist-led introduction to sport during inpatient rehabilitation	Single-investigator telephone questionnaire survey	33/39 (84.6%) patients contacted; 14 reported regular sporting activity after discharge; of those regularly exercising, 60% had also exercised prediagnosis (continuation), while 27% of prior non-exercisers newly took up sport after injury (new entry); most active patients were male and aged 16-35	4 Quantitative descriptive	≤2/5
109	Kars, Hofman, Geertzen, Pepping & Dekker, 2009	Netherlands	Quantitative descriptive (survey)	105	Lower limb amputation	N/R	N/R	Not sport-specific (recreational activity/sport participation habits)	Entry/Initiation	None stated		Prior (pre-amputation) participation associated with post-amputation sport participation (implied)	Self-constructed questionnaire	105 lower limb amputees responded (36% response rate); 32% participated in some form of sport; participation appeared linked to pre-amputation activity history	4 Quantitative descriptive	3/5
110	Muselaers, van Eck, van Schie, Boeker & Buizer, 2026	Netherlands	Qualitative	49	Physical disability (children)	8–71 years across stakeholder groups	67.3% (33/49 across stakeholder groups)	Regular (mainstream) sport clubs	Entry/Initiation	Child-centered approach (methodological/philosophical framing, not a formal psychological theory)		Regular sport club context and structures examined for enhancing inclusion of children with physical disability	Qualitative interviews (child-centered methodology)	Explored how a child-centered approach can enhance participation of children with physical disability in regular (mainstream) sport clubs	1 Qualitative	≥4/5
111	Storli, Aune & Lorås, 2022	Norway	Qualitative (life-history/developmental)	8	Physical disability (elite/world-class para-athletes)	N/R	N/R	Multiple (World Para Athletics classes; top-ranked athletes)	Entry/Initiation; Observed Behavioral Persistence	Athlete developmental pathways framework		Heterogeneity of disability type/degree as a factor diversifying developmental pathways compared to able-bodied athletes		Investigated developmental pathways of 8 top-ranked para-athletes toward elite/world-class performance, hypothesizing pathways would be more diversified than for able-bodied athletes due to heterogeneity in disability type and degree	1 Qualitative	3/5
112	Berthiaume, Ouellet, Labbé, Hebinck, Robert, Descarreaux, Martineau & Best, 2025	Canada	Qualitative	9	Physical disability (general)	31–60 years (median 40)	88.9% (8/9)	Adapted paddleboarding	Entry/Initiation	None stated (Quality of Participation framework possibly implied by title)	Meaningful engagement in the adapted sport program	Program-level strategies to support quality of participation		Explored the quality of participation and identified strategies to support meaningful engagement in an adapted paddleboard program for individuals with disabilities	1 Qualitative	3/5
113	Al Harthy, Hammad & Awed, 2024	Saudi Arabia	Quantitative descriptive (cross-sectional)	205	Physical disability (people with disabilities, general)	N/R	N/R	Sport (general, not sport-specific)	Current/General Participation	None stated	Motivation examined as a function of age, gender, disability type, and educational level	Educational level and disability type as socio-demographic contextual factors	Cross-sectional survey (motivation instrument not fully named in visible excerpt)	Determined motivation for sports participation among 205 people with disabilities in Saudi Arabia, considering age, gender, disability type, and educational level as influencing factors, framed within sport's role in social integration	4 Quantitative descriptive	3/5
114	Tóth & Máté, 2025	Hungary	Mixed methods	122	Physical disability (76 athletes among sample)	N/R	N/R	Multiple (reduced-mobility sports)	Current/General Participation	None stated (intrinsic/extrinsic motivation framing)	Sports motivation (gender found unrelated to motivation, despite affecting participation)	Extrinsic factors particularly salient for athletes with reduced mobility; expert (sports-leader) perspectives on opportunity structures	Questionnaire (quantitative); expert interviews with sports leaders (qualitative)	Examined sports motivation and opportunities for people with physical disability in Hungary; gender affected participation but not motivation; extrinsic factors were particularly important for athletes with reduced mobility	5 Mixed methods	≤2/5
115	Top & Akil, 2021	Türkiye	Quantitative descriptive (cross-sectional)	688	Physical disability (various types, active in sport)	N/R	N/R	Multiple (various sports, various regions of Turkey)	Current/General Participation	None stated	Motivation level (Motivation Scale for Sports Participation) linked to prosocial behavior	Type of disability examined as a moderator of motivation and prosocial/antisocial behavior	Motivation Scale for Sports Participation of People with Disabilities (MSSPPD); prosocial/antisocial behavior scale	Among 688 Turkish individuals with physical disabilities who do sports, motivation levels were related to prosocial and antisocial behaviors; disability type was examined as a potential moderating factor	4 Quantitative descriptive	≤2/5
116	Connor-Kuntz, Dummer & Paciorek, 1995	USA	Quantitative descriptive	133	Spina bifida (myelomeningocele)	7-16 years	N/R	Sport (general) and	Current/General Participation	None stated	Not the primary focus (structural/access factors are the focus)	Age (elementary-aged least likely excluded from PE); ambulation status (full-time	Researcher-developed survey (PE and sport participation questionnaire)	Among 133 children/youth with myelomeningocele (7-16y), 90.2% received physical	4 Quantitative descriptive	≤2/5

No.	Author(s), Year	Country	Study Design	Sample Size (N)	Disability Type	Age Range	% Female	Sport Type/Level	Participation Process / Evidence Category	Theoretical Framework	Motivational Factors	Contextual Factors	Measurement Instruments	Key Findings	MMAT Category	Consensus MMAT Criteria Met (descriptive only)
								physical education				manual wheelchair users least likely excluded); MM level (cervical MM associated with lower participation); PE placement often based on diagnostic label rather than actual ambulation ability		education (51.7% in regular PE placements), and 82.6% reported sport participation; participation varied by age, MM level, and ambulation status, with placement sometimes based on diagnostic label rather than functional ability		
117	Osmotherly, Thompson, Rivett, Haskins & Snodgrass, 2021	Australia	Quantitative descriptive (cross-sectional)	71	Physical disability (wheelchair sports participants)	mean 47.7 years (SD 16.4)	21.1% (15/71)	Wheelchair sports (community-based, multiple)	Current/General Participation	None stated		Injury prevalence/management as a barrier to continued participation; benefits and barriers to wheelchair sports participation examined		Examined injuries, practices, and perceptions of Australian wheelchair sports participants; long-term injury management and its impact on participation was a key focus	4 Quantitative descriptive	≥4/5
118	Koper, Rozmiarek, Poczta, Malchrowicz-Mośko & Tasiemski, 2024	Poland	Quantitative (comparative)	119	Physical disability (mass running event participants)	mean 36 years	48.7%	Mass running events (wheelchair and standing/ambulatory modes)	Current/General Participation	None stated	Motives for participation compared by sex, age group, and time since injury/illness	Mode of movement during run (wheelchair vs. standing) as a structural/physical factor		Identified motives for participation in mass running events among people with physical disabilities, comparing by sex, age group (emerging vs. middle-aged adults), time since injury/illness, and movement mode (wheelchair vs. standing)	4 Quantitative descriptive	≤2/5
119	Jeong & Park, 2013	South Korea	Quantitative descriptive (comparative)	66	Physical disability (wheelchair tennis players)	N/R	N/R	Wheelchair tennis (2013 Korea Open Wheelchair Tennis Tournament)	Current/General Participation	None stated	Enjoyment motivation (higher among Korean players); learning, health-fitness, and enjoyment motivation (negatively correlated with physical anxiety among Korean players)	Nationality/cultural context (Korean vs. non-Korean) as a comparative factor; competitive tournament setting	Participation Motivation Questionnaire; competition anxiety measures	Among 66 wheelchair tennis players, Korean players reported significantly higher enjoyment motivation, while non-Korean players showed higher cognitive anxiety and self-confidence; Korean players' physical anxiety was negatively correlated with learning, health-fitness, and enjoyment motivation	4 Quantitative descriptive	≤2/5
120	Kunene, 2025	South Africa	Qualitative	23	Physical disability (Paralympians)	mean 26 years	52.2% (12/23)	Multiple (Paralympic sports)	Current/General Participation	None stated	Passion, desire, fun, enjoyment (facilitators, per literature review framing)	Low- and middle-income country context; transformation initiatives insufficient; sporting-code-specific access via SASAPD (South African Sports Association for Physically Disabled)	Semi-structured interviews	Explored barriers, facilitators, and needs of South African Paralympians, highlighting that athletes with disabilities in low-/middle-income countries like South Africa continue to face significant challenges despite transformation initiatives	1 Qualitative	3/5
121	Merovitz-Budning, Wexler, Vance, Dam, Yan, Saba, Coulter, Lemay, Musselman & Cheung, 2025	Canada	Quantitative descriptive (cross-sectional survey)	72	Spinal cord injury or disease (aged 50+)	≥50 years	30.6% (22/72)	Adaptive sport (general, incl. sledge hockey)	Withdrawal/Dropout/Nonparticipation	None stated	Satisfaction with physical, social (and presumably psychological) domains among participants	Demographic/access factors: gender, race, age, urban/rural residence, income, employment status, transportation access; injury factors: paraplegia vs. tetraplegia, motor-incomplete vs. complete injury	Researcher-developed survey (descriptive cross-sectional)	Compared adults aged 50+ with SCI/D who do vs. do not participate in adaptive sport; participants were hypothesized (based on prior under-50 literature) to more often be male, white, younger, urban, higher-income, employed, have reliable transportation, have paraplegia/motor-incomplete injury, and report greater satisfaction	4 Quantitative descriptive	≥4/5
122	Bragaru, van Wilgen, Geertzen, Ruijs, Dijkstra & Dekker, 2013	Netherlands	Qualitative	26	Lower limb amputation (13 of 26 were athletes)	N/R	N/R	Multiple (general sport participation, LLA)	Withdrawal/Dropout/Nonparticipation	None stated	Increased social contacts; better self-esteem and body-image among sport participants (from literature review framing, examined qualitatively)		Semi-structured interviews; thematic analysis	Fewer than 40% of individuals with lower limb amputation participate in sports; this qualitative study of 26 individuals (13 athletes) from Groningen/Drenthe identified barriers and facilitators to sports participation via semi-structured interviews and thematic analysis	1 Qualitative	≥4/5

N/R = Not reported in the original publication.
