# Supplementary material for: Motivational and Contextual Factors Across Sport Entry, Current Participation, Maintenance, and Withdrawal Among People with Physical Disabilities: A Systematic Review
Source: Sports (Basel). 2026 Oct 14;14(10):457. doi: 10.3390/sports14100457 (PMC13709342; doi:10.3390/sports14100457)
Supplement: Supplementary file 1 [file sports-14-00457-s001.zip › Table_S3_Decision_Rules.pdf]

Table S3. Operational decision rules for participation-process and maintenance-evidence coding

Motivational and Contextual Factors Across Sport Entry, Current Participation, Maintenance, and Withdrawal Among People with Physical Disabilities: A Systematic Review

These decision rules were developed during the internal data-verification process and applied conservatively by both reviewers. The framework is not intended to represent four strictly sequential temporal stages. Current/General Participation is a cross-sectional evidence category, whereas Entry/Initiation, Maintenance-Related Evidence, and Withdrawal/Dropout/Nonparticipation refer more directly to participation processes. Maintenance-related studies were further distinguished according to whether they assessed Indirect/Prospective Indicators of Continuation or Provided Evidence of Observed Behavioral Persistence. Studies could be coded into more than one category when a single design directly addressed multiple participation processes.

Participation Process / Evidence Category	Operational Decision Rule
Entry/Initiation	The study directly addresses the decision, event, or process of beginning sport participation, including motives for initial engagement, explicit sport initiation or introduction, pre- versus post-disability-onset participation, or structured introductory/taster programs intended to facilitate entry into sport.
Current/General Participation	The study describes psychological, motivational, interpersonal, or contextual characteristics of individuals who are currently participating in sport at a single time point but provides no direct evidence regarding whether participation has recently begun, is being sustained over time, or is at risk of discontinuation. This is a cross-sectional evidence category and is used when no entry-, maintenance-, or withdrawal-specific criterion is met.
Indirect/Prospective Maintenance-Related Evidence	The study examines constructs relevant to continued participation without directly demonstrating that sport participation was behaviorally sustained over time. Indicators may include intention to continue, sport commitment, perceived future participation, sustained interest, or perceived facilitators/barriers to continuation. These indicators are interpreted as maintenance-related evidence, not as observed persistence.
Observed Behavioral Persistence	The study provides evidence that sport participation itself was sustained across time through longitudinal follow-up, repeated participation measurement, documented retention, or clearly documented retrospective continuation. Retrospective/life-history evidence is distinguished descriptively from prospectively observed longitudinal persistence in the synthesis.
Withdrawal/Dropout/Nonparticipation	The study directly examines discontinuation, dropout, non-uptake, or nonparticipation, or explicitly compares participants with non-participants. Studies may be coded here whether nonparticipants previously participated and stopped or never initiated sport participation, provided that this status is clearly reported.
