# Supplementary material for: Motivational and Contextual Factors Across Sport Entry, Current Participation, Maintenance, and Withdrawal Among People with Physical Disabilities: A Systematic Review
Source: Sports (Basel). 2026 Oct 14;14(10):457. doi: 10.3390/sports14100457 (PMC13709342; doi:10.3390/sports14100457)
Supplement: Supplementary file 1 [file sports-14-00457-s001.zip › Table_S4_Detailed_MMAT.pdf]

Table S4. Detailed MMAT quality appraisal of included studies (n = 106)

Motivational and Contextual Factors Across Sport Entry, Current Participation, Maintenance, and Withdrawal Among People with Physical Disabilities: A Systematic Review

GE-1 to GE-5 = G.E.B.'s independent ratings on the five MMAT criteria for the study's design category; UA-1 to UA-5 = U.A.'s independent ratings; C-1 to C-5 = final consensus rating for each criterion. Y = Yes, N = No, CT = Cannot tell. Rows highlighted in yellow indicate at least one criterion on which the two reviewers' independent ratings initially differed (34 of the final 106 included studies; inter-rater agreement prior to consensus: raw agreement = 92.3%, Cohen's κ = 0.84 — Section 3.3). All disagreements were resolved through consensus discussion (Section 2.6); where discussion did not fully converge, the more conservative rating was adopted (No > Cannot tell > Yes). The consensus ratings (C-1 to C-5) determine the overall MMAT category and the descriptive count of criteria met (≤2/5, 3/5, ≥4/5) reported in Table S2; this count is presented for descriptive purposes only and is not an MMAT-endorsed composite quality score.

No.	Author(s), Year	MMAT Category	GE-1	GE-2	GE-3	GE-4	GE-5	UA-1	UA-2	UA-3	UA-4	UA-5	C-1	C-2	C-3	C-4	C-5
14	McLoughlin, Weisman Fecske, Castaneda, Gwin & Graber, 2017	1 Qualitative	Y	Y	Y	Y	Y	Y	Y	Y	Y	Y	Y	Y	Y	Y	Y
18	Severin, Kinderen & Baumgart, 2025	3 Non-randomized	Y	CT	CT	CT	CT	Y	CT	CT	CT	CT	Y	CT	CT	CT	CT
19	Yilmaz, Kirimoglu & Mirze, 2020	4 Quantitative descriptive	Y	N	Y	CT	N	Y	N	Y	CT	N	Y	N	Y	CT	N
20	Ben Rakaa, Lourenço, Bassiri & Lotfi, 2025	2 Randomized controlled trials	CT	Y	Y	CT	Y	CT	Y	Y	CT	Y	CT	Y	Y	CT	Y
21	Tasiemski & Brewer, 2011	4 Quantitative descriptive	CT	Y	Y	Y	Y	Y	Y	Y	Y	Y	CT	Y	Y	Y	Y
22	Tasiemski, Kennedy, Gardner & Blaikley, 2004	4 Quantitative descriptive	CT	CT	Y	Y	Y	Y	CT	Y	Y	Y	CT	CT	Y	Y	Y
23	Maciá-Andreu, Vaquero-Cristóbal, Meroño, Abenza-Cano, García-Roca, Cánovas-Álvarez, Díaz-Aroca, Capranica, Stanescu, Pereira, Doupona, Mendes, Figueiredo, Isidori, Sánchez-Pato & Leiva-Arcas, 2023	4 Quantitative descriptive	CT	CT	Y	CT	Y	CT	CT	Y	CT	Y	CT	CT	Y	CT	Y
24	Perrier, Sweet, Strachan & Latimer-Cheung, 2012	3 Non-randomized	Y	Y	Y	CT	CT	Y	Y	Y	CT	CT	Y	Y	Y	CT	CT
25	Mulligan, Miyahara & Nichols-Dunsmuir, 2017	1 Qualitative	Y	Y	Y	Y	Y	Y	Y	Y	CT	CT	Y	Y	Y	CT	CT
26	Castelli Correia de Campos, Fica, Fernandéz, Teixeira Fabricio dos Santos, Gamonales, Winckler, Pena, Muñoz, Pleticosic, Luarte Rocha, Rossato de Victo, del Val Martín & Ferrari, 2025	4 Quantitative descriptive	CT	CT	Y	CT	Y	Y	CT	Y	CT	Y	CT	CT	Y	CT	Y
27	Leung, Chung & Chu, 2020	1 Qualitative	Y	Y	Y	Y	Y	Y	Y	Y	Y	Y	Y	Y	Y	Y	Y
28	Aytur, Craig, Frye, Bonica, Rainer, Hapke & McGilvray, 2018	1 Qualitative	Y	Y	Y	Y	CT	Y	Y	Y	CT	CT	Y	Y	Y	CT	CT
29	Epiney, Hany, Nagel & Klenk, 2026	4 Quantitative descriptive	Y	Y	Y	Y	Y	Y	Y	Y	Y	Y	Y	Y	Y	Y	Y
30	Shirazipour, Latimer-Cheung & Arbour-Nicitopoulos, 2015	1 Qualitative	Y	Y	Y	Y	Y	Y	Y	Y	Y	Y	Y	Y	Y	Y	Y
31	Perrier, Shirazipour & Latimer-Cheung, 2015	4 Quantitative descriptive	Y	Y	Y	CT	Y	Y	Y	Y	CT	Y	Y	Y	Y	CT	Y
32	Perrier, Latimer-Cheung, Martin Ginis & SHAPE-SCI Research Team, 2012	3 Non-randomized	Y	Y	CT	Y	CT	Y	Y	CT	Y	CT	Y	Y	CT	Y	CT
33	Stapleton, Perrier, Campbell, Tawse & Martin Ginis, 2016	3 Non-randomized	CT	Y	Y	CT	CT	CT	Y	Y	CT	CT	CT	Y	Y	CT	CT
34	Jaarsma, Dijkstra, de Blécourt, Geertzen & Dekker, 2015	5 Mixed methods	Y	Y	CT	CT	CT	Y	Y	Y	CT	CT	Y	Y	CT	CT	CT
35	Jaarsma, Dekker, Geertzen & Dijkstra, 2016	4 Quantitative descriptive	CT	Y	Y	CT	Y	Y	Y	Y	CT	Y	CT	Y	Y	CT	Y
36	Nam, Lee, Jun, Parke, Kim & Chae, 2016	4 Quantitative descriptive	Y	CT	Y	CT	CT	Y	Y	Y	CT	CT	Y	CT	Y	CT	CT
37	Yao & Shapiro, 2024	3 Non-randomized	CT	Y	CT	CT	CT	CT	Y	CT	CT	CT	CT	Y	CT	CT	CT
38	Martin, Byrd, Watts & Dent, 2015	4 Quantitative descriptive	CT	CT	Y	Y	Y	Y	CT	Y	Y	Y	CT	CT	Y	Y	Y
39	Atkinson & Martin, 2020	3 Non-randomized	CT	Y	Y	Y	CT	CT	Y	Y	Y	CT	CT	Y	Y	Y	CT
40	Buffart, van der Ploeg, Bauman, van Asbeck, Stam, Roebroeck & van den Berg-Emons, 2008	4 Quantitative descriptive	Y	CT	Y	CT	Y	Y	CT	Y	CT	Y	Y	CT	Y	CT	Y
41	Bogado & Villalba, 2023	5 Mixed methods	Y	CT	CT	CT	CT	Y	CT	CT	CT	CT	Y	CT	CT	CT	CT
42	Clutterbuck, Auld & Johnston, 2022	5 Mixed methods	Y	CT	CT	CT	CT	Y	CT	CT	CT	CT	Y	CT	CT	CT	CT
43	Blinde & McCallister, 1999	1 Qualitative	Y	Y	Y	Y	Y	Y	CT	Y	CT	CT	Y	CT	Y	CT	CT
44	Vila-Nova, Santos, João & Pascoal, 2026	1 Qualitative	Y	Y	Y	Y	Y	Y	Y	Y	Y	Y	Y	Y	Y	Y	Y
45	Day, 2013	1 Qualitative	Y	CT	Y	Y	CT	Y	CT	Y	Y	CT	Y	CT	Y	Y	CT

No.	Author(s), Year	MMAT Category	GE-1	GE-2	GE-3	GE-4	GE-5	UA-1	UA-2	UA-3	UA-4	UA-5	C-1	C-2	C-3	C-4	C-5
46	Clutterbuck, Auld & Johnston, 2022	2 Randomized controlled trials	CT	CT	CT	Y	Y	Y	CT	CT	Y	Y	CT	CT	CT	Y	Y
47	Alarfaj, Hassan, Aljohar, Almuaili & Hassan, 2025	1 Qualitative	Y	Y	Y	Y	Y	Y	Y	Y	Y	Y	Y	Y	Y	Y	Y
48	Yılmaz, Gümüşay & Akkaya, 2021	4 Quantitative descriptive	CT	CT	Y	CT	Y	Y	CT	Y	CT	Y	CT	CT	Y	CT	Y
49	Ballas, Buultjens, Murphy & Jackson, 2022	1 Qualitative	Y	Y	Y	Y	Y	Y	Y	Y	Y	Y	Y	Y	Y	Y	Y
50	Orr, Tamminen, Sweet, Tomasone & Arbour-Nicitopoulos, 2018	1 Qualitative	Y	CT	Y	Y	CT	Y	CT	Y	Y	CT	Y	CT	Y	Y	CT
51	Sousa Junior, Clutterbuck, Guimaraes, Souza, Silva, Wright, Camargos & Leite, 2026	2 Randomized controlled trials	CT	CT	CT	CT	CT	Y	CT	CT	CT	CT	CT	CT	CT	CT	CT
52	Freitas, Oliveira, Arruda, Oliveira & Oliveira, 2021	4 Quantitative descriptive	CT	CT	Y	CT	Y	Y	CT	Y	CT	Y	CT	CT	Y	CT	Y
53	Asare, Townsend & Burrows, 2023	1 Qualitative	Y	Y	Y	Y	Y	Y	Y	Y	Y	Y	Y	Y	Y	Y	Y
54	Hernández-Lanas, Bruna-Torres, Carrasco-Palacios, Lucero-Sánchez & Molina-Pacheco, 2024	1 Qualitative	Y	Y	Y	CT	CT	Y	Y	Y	CT	CT	Y	Y	Y	CT	CT
55	Cheung, Buren, Benn, Alton, Craven, Marzolini & Musselman, 2025	5 Mixed methods	Y	Y	Y	Y	CT	Y	Y	Y	Y	CT	Y	Y	Y	Y	CT
56	Sarol, 2024	1 Qualitative	Y	Y	Y	Y	Y	Y	Y	Y	Y	Y	Y	Y	Y	Y	Y
57	Bates, Kearns, Witten & Carroll, 2019	1 Qualitative	Y	Y	Y	CT	CT	Y	Y	CT	CT	CT	Y	Y	CT	CT	CT
58	Cheung, Benn, Buren & Musselman, 2026	1 Qualitative	Y	Y	Y	Y	Y	Y	Y	Y	Y	Y	Y	Y	Y	Y	Y
59	van Oorschot, van Ee & Keijsers, 2023	3 Non-randomized	CT	Y	Y	CT	CT	CT	Y	Y	CT	Y	CT	Y	Y	CT	CT
60	Clutterbuck, Gent & Thomson, 2025	1 Qualitative	Y	Y	Y	CT	CT	Y	Y	Y	CT	CT	Y	Y	Y	CT	CT
61	Javorina, Shirazipour, Allan & Latimer-Cheung, 2020	1 Qualitative	Y	Y	Y	CT	CT	Y	Y	Y	CT	CT	Y	Y	Y	CT	CT
62	Kong, Quek, Liu, Ma & Tan, 2026	5 Mixed methods	CT	Y	CT	CT	CT	CT	Y	CT	CT	CT	CT	Y	CT	CT	CT
63	Amberkar, Agarwal, Singh, Shete & Mullerpatan, 2019	4 Quantitative descriptive	Y	CT	CT	Y	Y	Y	CT	Y	Y	Y	Y	CT	CT	Y	Y
64	Ćorić & Ljubotina, 2013	4 Quantitative descriptive	CT	CT	Y	CT	Y	CT	CT	Y	CT	Y	CT	CT	Y	CT	Y
65	Prokopowicz, Molik, Prokopowicz, Chamera, Ogonowska-Słodownik, Lencse-Mucha, Morgulec-Adamowicz, Kosmol & Perkowski, 2016	4 Quantitative descriptive	CT	CT	Y	CT	Y	CT	CT	Y	CT	Y	CT	CT	Y	CT	Y
66	Swanson, Colwell & Zhao, 2008	4 Quantitative descriptive	Y	CT	Y	CT	Y	Y	CT	Y	CT	Y	Y	CT	Y	CT	Y
67	Field & Oates, 2001	4 Quantitative descriptive	CT	CT	Y	CT	CT	CT	CT	Y	CT	CT	CT	CT	Y	CT	CT
68	Namai, Jung, Shaw & Martin Ginis, 2025	1 Qualitative	Y	Y	Y	Y	Y	Y	Y	Y	Y	Y	Y	Y	Y	Y	Y
69	Ali & Rider, 2024	1 Qualitative	Y	Y	Y	Y	Y	Y	Y	Y	Y	Y	Y	Y	Y	Y	Y
70	Moll & Bester, 2019	4 Quantitative descriptive	CT	CT	Y	CT	Y	CT	CT	Y	CT	Y	CT	CT	Y	CT	Y
71	Sit, Lindner & Sherrill, 2002	4 Quantitative descriptive	Y	CT	Y	Y	Y	Y	CT	Y	Y	Y	Y	CT	Y	Y	Y
72	Tasiemski, Bergström, Savic & Gardner, 2000	4 Quantitative descriptive	CT	CT	Y	CT	Y	Y	CT	Y	CT	Y	CT	CT	Y	CT	Y
73	Qi, Cai & Meng, 2021	1 Qualitative	Y	Y	Y	Y	Y	Y	Y	Y	Y	Y	Y	Y	Y	Y	Y
74	Coates & Vickerman, 2016	1 Qualitative	Y	CT	Y	Y	CT	Y	CT	Y	Y	CT	Y	CT	Y	Y	CT
75	Ojwang, Foster & Odiango, 2025	1 Qualitative	Y	Y	Y	Y	Y	Y	Y	CT	Y	CT	Y	Y	CT	Y	CT
76	Hayward, Hogan, Melam, Raine, McCullough & Bell, 2025	5 Mixed methods	Y	Y	CT	CT	CT	CT	Y	CT	CT	CT	CT	Y	CT	CT	CT
77	Kim, Thomas, Sharpe & Farrell, 2025	1 Qualitative	Y	Y	Y	Y	Y	Y	Y	Y	Y	Y	Y	Y	Y	Y	Y
78	Kim, Kang & Seo, 2005	4 Quantitative descriptive	Y	CT	CT	Y	Y	Y	CT	CT	Y	Y	Y	CT	CT	Y	Y
79	Feiten, Schaab & Sanfelice, 2025	1 Qualitative	Y	Y	Y	Y	CT	Y	Y	Y	CT	CT	Y	Y	Y	CT	CT
80	Ortiz Cárdenas, Perdomo Vargas, Garzón Pastrana & Vargas Reyes, 2021	4 Quantitative descriptive	CT	CT	Y	CT	Y	Y	CT	Y	CT	Y	CT	CT	Y	CT	Y
81	Stephens, Neil & Smith, 2012	1 Qualitative	Y	Y	Y	Y	Y	Y	Y	CT	Y	CT	Y	Y	CT	Y	CT
82	Sá, Azevedo, Martins, Machado & Tavares, 2012	4 Quantitative descriptive	CT	CT	Y	CT	CT	Y	CT	Y	CT	CT	CT	CT	Y	CT	CT
83	Comaduran Marquez, Vaandering, Babwani, Redquest, Nikitovic, Kelly, Kinney-Lang & Kirton, 2026	5 Mixed methods	Y	Y	CT	CT	CT	Y	Y	CT	CT	CT	Y	Y	CT	CT	CT
84	Barbosa de Lima & de Jesus Alves, 2020	1 Qualitative	Y	CT	CT	CT	CT	CT	CT	CT	CT	CT	CT	CT	CT	CT	CT
85	Berardi, Galeoto, Lucibello, Panuccio, Valente & Tofani, 2021	4 Quantitative descriptive	Y	CT	Y	CT	Y	Y	CT	Y	CT	Y	Y	CT	Y	CT	Y

No.	Author(s), Year	MMAT Category	GE-1	GE-2	GE-3	GE-4	GE-5	UA-1	UA-2	UA-3	UA-4	UA-5	C-1	C-2	C-3	C-4	C-5
86	Hernández-Lanas, Molina-Pacheco, Lucero-Sánchez, Carrasco-Palacios & Bruna-Torres, 2026	4 Quantitative descriptive	CT	CT	Y	CT	CT	CT	CT	Y	CT	CT	CT	CT	Y	CT	CT
87	Poonsiri, Dekker, Dijkstra, Hijmans & Geertzen, 2021	4 Quantitative descriptive	Y	CT	Y	Y	Y	Y	CT	Y	Y	Y	Y	CT	Y	Y	Y
88	Bragaru, Meulenbelt, Dijkstra, Geertzen & Dekker, 2013	4 Quantitative descriptive	CT	Y	Y	Y	Y	Y	Y	Y	Y	Y	CT	Y	Y	Y	Y
89	Nettleton, Hassett, Scheibe, Price, Kirkham & Sherrington, 2017	3 Non-randomized	CT	Y	Y	CT	CT	CT	Y	Y	CT	CT	CT	Y	Y	CT	CT
90	Ayyildiz, Aras, Yagin, Gülü, Yapici & Alwhaibi, 2024	4 Quantitative descriptive	CT	CT	Y	CT	Y	Y	CT	Y	CT	Y	CT	CT	Y	CT	Y
91	Dutia, Curran, Donohoe, Beckman & Tweedy, 2022	3 Non-randomized	CT	Y	CT	Y	CT	CT	Y	CT	Y	CT	CT	Y	CT	Y	CT
92	Scheuer, Anneken, Hirschfeld, Richarz & Thietje, 2009	4 Quantitative descriptive	CT	Y	Y	CT	Y	CT	Y	Y	CT	Y	CT	Y	Y	CT	Y
93	Faraji, Dabiri, Allami, Hosseini & Barati, 2023	3 Non-randomized	CT	Y	CT	Y	CT	CT	Y	CT	Y	CT	CT	Y	CT	Y	CT
94	Çelenk, 2021	4 Quantitative descriptive	CT	CT	Y	CT	Y	Y	CT	Y	CT	Y	CT	CT	Y	CT	Y
95	Tekkurşun-Demir & İlhan, 2020	4 Quantitative descriptive	CT	CT	Y	CT	Y	CT	CT	Y	CT	Y	CT	CT	Y	CT	Y
96	Pérez-Tejero, Borges, Chávez, Pérez-Rodríguez & Coterón, 2023	4 Quantitative descriptive	CT	CT	Y	CT	Y	Y	CT	Y	CT	Y	CT	CT	Y	CT	Y
97	Guven, Kara & Ozdedeoglu, 2019	1 Qualitative	Y	Y	Y	CT	CT	Y	Y	Y	CT	CT	Y	Y	Y	CT	CT
98	Alhumaid, Adnan & Said, 2024	1 Qualitative	Y	CT	Y	Y	CT	Y	CT	Y	Y	CT	Y	CT	Y	Y	CT
99	Anderson, Wozencroft & Bedini, 2008	4 Quantitative descriptive	Y	CT	Y	Y	CT	Y	CT	Y	Y	CT	Y	CT	Y	Y	CT
100	Darcy, Lewis, Fausett, Neuville, Bennett & Jayabalan, 2024	4 Quantitative descriptive	CT	Y	Y	CT	Y	CT	Y	Y	CT	Y	CT	Y	Y	CT	Y
101	Akhoroz, Erdogan & Yildirim, 2025	1 Qualitative	Y	Y	Y	Y	Y	Y	Y	Y	Y	Y	Y	Y	Y	Y	Y
102	Hudson, Sanzo, Zerpa & Klarner, 2024	4 Quantitative descriptive	Y	CT	Y	Y	Y	Y	CT	Y	Y	Y	Y	CT	Y	Y	Y
103	Samsonienė, Adomaitienė, Krivičiūtė, Jankauskienė, Jurkštienė & Kėvelaitis, 2008	4 Quantitative descriptive	CT	CT	Y	CT	Y	CT	CT	Y	CT	Y	CT	CT	Y	CT	Y
104	Schenker, Wolf, Alimusaj, Putz, Lehner & Block, 2025	3 Non-randomized	CT	Y	CT	CT	CT	CT	Y	CT	CT	CT	CT	Y	CT	CT	CT
105	Furst, Ferr & Megginson, 1993	4 Quantitative descriptive	CT	CT	Y	Y	Y	CT	CT	Y	Y	Y	CT	CT	Y	Y	Y
106	Wu & Williams, 2001	4 Quantitative descriptive	Y	CT	Y	Y	Y	Y	CT	Y	Y	Y	Y	CT	Y	Y	Y
107	Kirkby, Cull & Foreman, 1996	4 Quantitative descriptive	Y	CT	CT	CT	Y	Y	CT	CT	CT	Y	Y	CT	CT	CT	Y
108	O'Neill & Maguire, 2004	4 Quantitative descriptive	CT	CT	Y	CT	CT	CT	CT	Y	CT	CT	CT	CT	Y	CT	CT
109	Kars, Hofman, Geertzen, Pepping & Dekker, 2009	4 Quantitative descriptive	CT	CT	Y	Y	Y	CT	CT	Y	Y	Y	CT	CT	Y	Y	Y
110	Muselaers, van Eck, van Schie, Boeker & Buizer, 2026	1 Qualitative	Y	Y	Y	Y	Y	Y	Y	Y	Y	Y	Y	Y	Y	Y	Y
111	Storli, Aune & Lorås, 2022	1 Qualitative	Y	Y	Y	CT	CT	Y	Y	Y	CT	CT	Y	Y	Y	CT	CT
112	Berthiaume, Ouellet, Labbé, Hebinck, Robert, Descarreaux, Martineau & Best, 2025	1 Qualitative	Y	Y	Y	Y	Y	Y	Y	CT	Y	CT	Y	Y	CT	Y	CT
113	Al Harthy, Hammad & Awed, 2024	4 Quantitative descriptive	Y	CT	Y	CT	Y	Y	CT	Y	CT	Y	Y	CT	Y	CT	Y
114	Tóth & Máté, 2025	5 Mixed methods	Y	Y	CT	CT	CT	Y	Y	CT	CT	CT	Y	Y	CT	CT	CT
115	Top & Akil, 2021	4 Quantitative descriptive	CT	CT	Y	CT	Y	Y	CT	Y	CT	Y	CT	CT	Y	CT	Y
116	Connor-Kuntz, Dummer & Paciorek, 1995	4 Quantitative descriptive	CT	CT	Y	Y	CT	Y	CT	Y	Y	CT	CT	CT	Y	Y	CT
117	Osmotherly, Thompson, Rivett, Haskins & Snodgrass, 2021	4 Quantitative descriptive	Y	CT	Y	Y	Y	Y	CT	Y	Y	Y	Y	CT	Y	Y	Y
118	Koper, Rozmiarek, Poczta, Malchrowicz-Mośko & Tasiemski, 2024	4 Quantitative descriptive	CT	CT	Y	CT	Y	Y	CT	Y	CT	Y	CT	CT	Y	CT	Y
119	Jeong & Park, 2013	4 Quantitative descriptive	CT	CT	Y	CT	Y	CT	CT	Y	CT	Y	CT	CT	Y	CT	Y
120	Kunene, 2025	1 Qualitative	Y	Y	Y	Y	Y	Y	Y	CT	Y	CT	Y	Y	CT	Y	CT
121	Merovitz-Budning, Wexler, Vance, Dam, Yan, Saba, Coulter, Lemay, Musselman & Cheung, 2025	4 Quantitative descriptive	Y	CT	Y	Y	Y	Y	CT	Y	Y	Y	Y	CT	Y	Y	Y
122	Bragaru, van Wilgen, Geertzen, Ruijs, Dijkstra & Dekker, 2013	1 Qualitative	Y	Y	Y	Y	Y	Y	Y	Y	Y	Y	Y	Y	Y	Y	Y
